# tidytcells: standardizer for TR/MH nomenclature

**DOI:** 10.3389/fimmu.2023.1276106

**Published:** 2023-10-25

**Authors:** Yuta Nagano, Benjamin Chain

**Affiliations:** ^1^ Division of Medicine, Faculty of Medical Scienecs, University College London (UCL), London, United Kingdom; ^2^ Division of Infection and Immunity, Faculty of Medical Sciences, University College London (UCL), London, United Kingdom

**Keywords:** T cell receptor (TCR), major histocompatibility complex (MHC), data preprocessing, data cleaning, python (programming language), T cell receptor repertoire

## Abstract

T cell receptors (TR) underpin the diversity and specificity of T cell activity. As such, TR repertoire data is valuable both as an adaptive immune biomarker, and as a way to identify candidate therapeutic TR. Analysis of TR repertoires relies heavily on computational analysis, and therefore it is of vital importance that the data is standardized and computer-readable. However in practice, the usage of different abbreviations and non-standard nomenclature in different datasets makes this data pre-processing non-trivial. tidytcells is a lightweight, platform-independent Python package that provides easy-to-use standardization tools specifically designed for TR nomenclature. The software is open-sourced under the MIT license and is available to install from the Python Package Index (PyPI). At the time of publishing, tidytcells is on version 2.0.0.

## 1 Introduction

**Figure 1:**
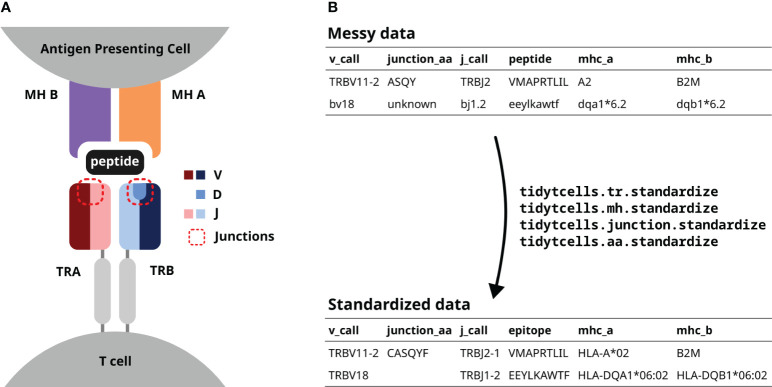
**(A)** A diagram of a TR interacting with a peptide-MH complex. The V, D and J genes comprising each TR chain are shown by color. The red dotted lines point out the junction sequences of both TR chains. **(B)** An illustration of how tidytcells can help clean TR data. By using tidytcells, non-standard nomenclature in the “messy data” is corrected, and any invalid values are filtered out.

T cells are an important immune cell population that help orchestrate the vertebrate adaptive immune system. They express T cell receptors (TR) on their cell surface (**Figure 1A**), which allows them to recognize and respond to antigens presented on the surfaces of other cells via the Major Histocompatibility (MH) proteins (1, 2). Each T cell clone has a specific antigenic stimulus that it can respond to, often termed a T cell’s “cognate antigen”. The great range of target specificity is made possible by the fact that each new T cell clone generates its own unique TR via a stochastic process of somatic gene rearrangement termed VDJ recombination.

Advances in high-throughput parallel sequencing allow large numbers of T cells isolated from blood or tissue to be sequenced. This gives us a snapshot of which T cells exist in the immune system of an individual at a given time, together with their frequency in the population, which is referred to as the individual’s TR repertoire. Because T cell clones proliferate after recognizing their cognate antigen, TR repertoire data has proven useful as an adaptive immune biomarker in various contexts, from cancer (3–6) to SARS-CoV-2 infection (7–10), and there is growing excitement that TR repertoire data can be used as a sensitive yet minimally invasive diagnostic biomarker for many other transmissible and non-transmissible diseases (11). TR repertoire data may also be exploited for therapeutic purposes, for example in the context of cellular therapies by contributing to the identification of TR with reactivities against clinically relevant targets (12, 13).

The TR is a heterodimer, created by imprecise somatic recombination of one of a set of V and J genes (alpha and gamma chains), or V, J and D genes (beta and delta chains) (**Figure 1A**). The current convention is to represent TR sequence data by specifying, for each of the two chains that comprise it, which variable (V) and joining (J) genes are used, and what the amino acid sequence is of the junction region (also known as the complementarity-determining region 3, or CDR3) between the V and J genes (**Figure 1B**). Because of junctional imprecision, this sequence is not template driven, and cannot be aligned to the germ line sequence. In many cases, for example where populations of cells are lysed before sequencing, alpha/gamma to beta/delta chain pairing is unresolved, and indeed only one chain (typically the TR beta) may be sequenced. In many studies, TR sequences are further annotated by their cognate peptide/MH (**Figure 1B**).

Although the immunology community has generally converged to a common format of TR data representation and has developed an international standardized nomenclature (14), TR data in practice still contain variation due to several issues. These include (**Figure 1B**):

- The use of non-standard TR/MH gene symbols
- The inclusion of non-functional TR genes when one is only interested in data for functional TR
- Differing levels of TR/MH gene resolution- for example, some data may resolve TR genes to the level of the allele (TRAV1-1*01) while others only to the level of the gene (TRAV1-1)

This variation is particularly problematic when trying to compile large sets of machine-readable TR for downstream computational analysis. For example, algorithms may not easily recognize that TRAV1-1 and TRAV1-1*01 are in fact the same TRAV. Similarly, HLA-A*01 may not be understood as semantically identical to the abbreviated symbol A1.

tidytcells is a lightweight python package that addresses this issue by providing simple-to-use utilities to standardize TR nomenclature. Its primary content is a set of functions that can convert non-standard TR/MH gene symbols into their international ImMunoGeneTics information system (IMGT)-standard versions (15). Additionally, it provides simple functions to standardize junction and epitope amino acid sequences, as well as some other extra utilities.

tidytcells is available on the Python Package Index(PyPI) at https://pypi.org/project/tidytcells/. The source code is available at https://github.com/yutanagano/tidytcells under the MIT license. For more details such as the API reference, please see the documentation at https://tidytcells.readthedocs.io/en/latest/.

## 2 Method (Software features)

We provide a high-level overview of tidytcells’ features below. For more detailed instructions on use, including API references for each function, please refer to the documentation page. The API references for each of the standardization functions also include an outline of their decision logic, so that users can make informed decisions about their scope and limitations. The reference files that tidytcells uses to determine which gene symbols are valid can be found in the **Supplementary Materials**, as well as the source code. At the time of publishing, tidytcells is at version 2.0.0.

### 2.1 TR gene symbol standardization

tidytcells provides the function tr.standardize, which takes as input a string representing a potentially non-standard TR gene symbol, and outputs the corresponding IMGT-standardized symbol.

By default, if the input string cannot be resolved to a known TR gene, the function outputs None. The function attempts to standardize to human TR genes by default, but *Mus musculus* genes are also supported. Further options can be specified to exclude non-functional TR genes, or limit the resolution of the symbols to the level of the gene (as opposed to allele). Below is a code block demonstrating the use of tr.standardize.

**Table:** 

>>> import tidytcells as tt >>> tt.tr.standardize (“aj1”) “TRAJ1” >>> tt.tr.standardize (“TRBV6-4*01”, precision=“gene”) “TRBV6-4” >>> result = tt.tr.standardize (“TRBV1”, enforce_functional=True) UserWarning: Failed to standardize “TRBV1” for species homosapiens: gene has no functional alleles. Attempted fix “TRBV1”. >>> print (result) None >>> tt.tr.standardize (“TCRBV22S1A2N1T”, species=“musmusculus”) “TRBV2”

### 2.2 MH gene symbol standardization

A similar function mh.standardize is available for standardizing MH gene symbols. Its function signature and behavior is essentially equivalent to its TR counterpart.

**Table:** 

>>> tt.mh.standardize (“HLA-A*01:01:01”, precision=“protein”) “HLA-A*01:01” >>> tt.mh.standardize (“CRW2”, species=“musmusculus”) “MH1-M5”

### 2.3 Junction/epitope amino acid sequence standardization

aa.standardize and junction.standardize provide standardization utilities for amino acid sequences. aa.standardize can be used to clean generic amino acid sequence data, including epitopes, while junction.standardize provides TR junction sequence-specific logic.

**Table:** 

>>> tt.junction.standardize (“sadaf”) “CSADAFF” >>> result = tt.junction.standardize (“sadaf”, strict=True) UserWarning: Input sadaf was rejected as it is not a valid junction sequence. >>> print (result) None

### 2.4 Extra utilities

A brief list of additional features provided by tidytcells is shown in **Table 1**.

**Table 1:** A brief overview of extra utilities provided by tidytcells.

Function	Description
mh.get_chain	Given an MH gene symbol, classify as alpha or beta chain
mh.get_class	Given an MH gene symbol, classify as MH1 or MH2
mh.query	Query the list of all known MH genes/alleles
tr.get_aa_sequence	Obtain the underlying amino acid sequence of a TR gene
tr.query	Query the list of all known TR genes/alleles

**Table 2:** Examples of standardization successes.

Category	Species	Input	Output
TR	*Homo sapiens*	TCRBV17S1	TRBV17
	*Homo sapiens*	TRAV15	TRAV15-1/DV6-1
	*Homo sapiens*	29/DV5*01	TRAV29/DV5*01
	*Homo sapiens*	TCRBV5-1*01 or TCRBV5-1*02	TRBV5-1*01
	*Mus musculus*	TCRAV14D-3/DV8*02&nbsp;	TRAV14D-3/DV8*02
MH	*Homo sapiens*	HLA-A*02:01 W167A mutant	HLA-A*02:01
	*Homo sapiens*	DQB1*06:02	HLA-DQB1*06:02
	*Homo sapiens*	B2M	B2M
	*Mus musculus*	H2-Q9	MH1-Q9
	*Mus musculus*	H2-Db	MH2-D1
Junction	N/A	AASANSGTYQR	CAASANSGTYQRF
	N/A	CSVNRDTGAGGYTF	CSVNRDTGAGGYTF
Epitope	N/A	VMAPRTLIL	VMAPRTLIL

**Table 3:** Examples of standardization failures.

Category	Species	Input	Reason for failure
TR	*Homo sapiens*	TCRAJ1-3	Nonexistent gene
	*Homo sapiens*	TRBV14DV4	Nonexistent gene
	*Mus musculus*	1	Insufficient information
	*Mus musculus*	12D-2	Insufficient information
	*Mus musculus*	TRVB13-1*02	Nonexistent gene
MH	*Homo sapiens*	HLA class II	Insufficient information
	*Homo sapiens*	HLA-DQ	Insufficient information
	*Homo sapiens*	human MR1 K43A mutant	Non-classical HLAs not supported
	*Mus musculus*	M23I	Mutation specifier with no gene
	*Mus musculus*	HLA-DRB1*04:01	Incorrect species annotation
Junction	N/A	IVRVSHN*G#RDNYGQNFV	Ambiguity symbols not supported
Epitope	N/A	LLFGFPVYV + SCM(F5)	Peptide modification not supported
	N/A	diclofenac	Not a peptide

## 3 Results (Application to real data)

As a test use case of tidytcells’ functionality, we used it in combination with the pandas package to clean TR and MH data from the Immune Epitope Database (IEDB) (16). Where species data was available on the database, it was used. For TR or MH samples missing species labels, the species *Homo sapiens* was assumed. Other settings were left at default values, and no particular restrictions on gene functionality were imposed. Standardization was considered a success if the species associated with a particular value was supported, and the function managed to resolve the value to a recognized IMGT-compliant symbol.

Out of 2225 unique TR gene symbol values found in the database, 2127 values (95.6%) were standardized successfully. Similarly, 173 of 284 MH genes (60.9%), 301,554 of 301,670 junction sequences (99.9%) and 1825 of 1996 epitopes (91.4%) were standardized. Some examples of standardization successes and failures are shown in **Tables 2** and **3**. The data and code used to obtain these results can be found as **Supplementary Materials**.

## 4 Discussion

As demonstrated, tidytcells in its current form can successfully standardize the majority of TR/MH data from public databases such as IEDB. However, there are still limitations to its standardization ability. Below we discuss current limitations that we as maintainers of tidytcells hope to address in the near future. The package is entirely open source and code contributions from the community are welcome.

Currently, when tidytcells encounters a string of the general form “A B” where A is a valid example of what it is attempting to standardize, it will ignore “B” and return “A” as the standardized form. This works well for cases like the first MH success example, where the B string is a qualifier string that can be removed without fundamentally changing the underlying data. However, in cases like the fourth TR success example where the string is of the form “A or B”, the intuitively better representation of the underlying data is to standardize to the greatest common factor of A and B (i.e. TRBV5-1 in this case). Implementing separate logic to handle these cases would improve standardization quality.

For junction sequence standardization, the default behavior when dealing with a valid amino acid sequence that does not start with a cysteine (C) and end with a phenylalanine (F) or tryptophan (W) is to append a “C” at the beginning and an “F” at the end, and return the resulting string. The logic is implemented this way because the most common reason for these missing residues is that some data sources encode the junction as the CDR3 sequence (without the starting C and ending F/W). However, this rudimentary logic always assumes that the junction terminates with an F rather than a W. A possible improvement would be to use prior knowledge of the amino acid sequences of J genes to better predict the terminal residue. It may also be useful to provide an option to perform the reverse procedure (i.e. remove the C and F/W residues).

Other areas of potential improvement include parsing amino acid ambiguity codes and peptide modification syntax, more optional standardization constraints (e.g. specify a-priori that values should be resolved to TRAV genes/alleles as opposed to any TR gene), support for non-classical MH, allele imputation (if a gene has only one allele, resolve to that allele), and support for more species (only *Homo sapiens* and *Mus musculus* are currently supported).

## 5 Conclusion

tidytcells is a lightweight Python package that solves the issue of messy TR/MH data by providing easy-to-use utilities for standardizing TR/MH gene symbols, as well as general and TR junction amino acid data. We believe this will prove to be a useful utility to the rapidly growing community of scientists who are studying the TR repertoire.

## Data availability statement

Publicly available datasets were analyzed in this study. This data can be found here: https://www.iedb.org/.

## Author contributions

YN: Conceptualization, Methodology, Software, Writing – original draft, Writing – review & editing. BC: Supervision, Writing – review & editing.
