# Supplementary material for: tidytcells: standardizer for TR/MH nomenclature
Source: Front Immunol. 2023 Oct 25;14:1276106. doi: 10.3389/fimmu.2023.1276106 (PMC10634431; doi:10.3389/fimmu.2023.1276106)
Supplement: Supplementary file 2 [file DataSheet_2.zip › test_iedb.pdf]

test_iedb

September 26, 2023

1 Practical test of standardiser coverage on IEDB data

1.1 Setup

[ ]:
```
import gzip
from itertools import product
import json
import pandas as pd
from pandas import isna
import tidytcells
```

[ ]:
```
with gzip.open("iedb_species_decoder.json.gz", "r") as f:
    species_decoder = json.load(f)
```

[ ]:
```
df = pd.read_csv("iedb.csv.zip")
df = df[df["Response Type"] == "T cell"]
```

```
/tmp/ipykernel_8270/1899971150.py:1: DtypeWarning: Columns (8,10,12,14,16,17,18,
19,20,21,22,23,24,25,26,31,32,37,38,45,46,47,48,49,50,51,52,53,54,60,61,66,67)
have mixed types. Specify dtype option on import or set low_memory=False.
  df = pd.read_csv("iedb.csv.zip")
```

[ ]:
```
df.head()
```

[ ]:
```
     Group Receptor ID  Receptor ID                             Reference IRI  \
145                 47           57  http://www.iedb.org/reference/1004539
146                 47           57  http://www.iedb.org/reference/1004539
147               8493           58  http://www.iedb.org/reference/1004580
148               8493           58  http://www.iedb.org/reference/1004580
149               8493           58  http://www.iedb.org/reference/1017865

                           Epitope IRI Description  \
145   http://www.iedb.org/epitope/69921   VMAPRTLIL
146   http://www.iedb.org/epitope/69921   VMAPRTLIL
147   http://www.iedb.org/epitope/16878   FLRGRAYGL
148  http://www.iedb.org/epitope/144889   FLRGRFYGL
149  http://www.iedb.org/epitope/142137   EEYLQAFTY
```

	Antigen
145	HLA class I histocompatibility antigen, Cw-3 a…
146	HLA class I histocompatibility antigen, Cw-3 a…
147	nuclear antigen EBNA-3
148	NaN
149	ATP-binding cassette sub-family D member 3

	Organism	Response Type
145	Homo sapiens (human)	T cell
146	Homo sapiens (human)	T cell
147	Human herpesvirus 4 (Epstein Barr virus)	T cell
148	NaN	T cell
149	Homo sapiens (human)	T cell

	Assay IDs	MHC Allele Names	…
145	1548960, 1583178	HLA-E*01:01, HLA-E*01:03	…
146	1583178	HLA-E*01:03	…
147	1814845, 1814846, 1814847	HLA-B8	…
148	1831737	HLA-B8	…
149	1778798	HLA-B*44:05	…

	Chain 2 CDR1 Start Curated	Chain 2 CDR1 End Curated
145	NaN	NaN
146	NaN	NaN
147	NaN	NaN
148	NaN	NaN
149	NaN	NaN

	Chain 2 CDR1 Start Calculated	Chain 2 CDR1 End Calculated
145	25.0	29.0
146	25.0	29.0
147	25.0	29.0
148	25.0	29.0
149	25.0	29.0

	Chain 2 CDR2 Curated	Chain 2 CDR2 Calculated	Chain 2 CDR2 Start Curated
145	NaN	FVKESK	NaN
146	NaN	FVKESK	NaN
147	NaN	FQNEAQ	NaN
148	NaN	FQNEAQ	NaN
149	NaN	FQNEAQ	NaN

	Chain 2 CDR2 End Curated	Chain 2 CDR2 Start Calculated
145	NaN	47.0
146	NaN	47.0
147	NaN	47.0
148	NaN	47.0

```
149                        NaN                            47.0

    Chain 2 CDR2 End Calculated
145                        52.0
146                        52.0
147                        52.0
148                        52.0
149                        52.0

[5 rows x 72 columns]
```

[ ]:
```
def rename_species(species):
    if type(species) != str:
        return pd.NA

    if "sapiens" in species.lower():
        return "homosapiens"

    if "musculus" in species.lower():
        return "musmusculus"

    return species


df["Chain 1 Species"] = df["Chain 1 Species"].map(
    lambda x: pd.NA if pd.isna(x) else species_decoder[str(int(x))]
)
df["Chain 1 Species"] = df["Chain 1 Species"].map(rename_species)
```

[ ]:
```
tr_gene_symbols = pd.concat(
    [
        df[[" ".join(parts), "Chain 1 Species"]].rename(
            columns={" ".join(parts): "gene", "Chain 1 Species": "species"}
        )
        for parts in product(
            ("Curated", "Calculated"), ("Chain 1", "Chain 2"), ("V Gene", "J␣
↪Gene")
        )
    ]
)
tr_gene_symbols = tr_gene_symbols[tr_gene_symbols["gene"].notna()].
↪drop_duplicates()
```

[ ]:
```
tr_gene_symbols
```

[ ]:
```
               gene              species
145      TRAV26-1*01          homosapiens
```

```
415       TRAV21*01          homosapiens
896     TRAV26-2*01          homosapiens
1524        TCRAV1-2          homosapiens
1835       TCRAV26-1          homosapiens
…                …                  …
27260     TRBJ2-7*01  synthetic construct
27270     TRBJ2-1*01  synthetic construct
38794     TRBJ2-6*01          musmusculus
67860     TRBJ2-5*01       Macaca mulatta
204155    TRBJ1-6*02        Gallus gallus

[2225 rows x 2 columns]
```

[ ]:
```python
junctions = pd.concat(
    [
        df[[" ".join(parts), "Chain 1 Species"]].rename(
            columns={" ".join(parts): "junction", "Chain 1 Species": "species"}
        )
        for parts in product(
            ("Chain 1", "Chain 2"), ("CDR3",), ("Calculated", "Curated")
        )
    ]
)
junctions = junctions[junctions["junction"].notna()].drop_duplicates()
```

[ ]:
```python
junctions
```

[ ]:
```
               junction      species
145         IVVRSSNTGKLI  homosapiens
187          AASANSGTYQR  musmusculus
190                  AAS  musmusculus
205          AALIQGAQKLV  homosapiens
321        AVRPLLDGTYIPT  homosapiens
…                    …          …
211479    CSVNRDTGAGGYTF         <NA>
211480     CSVRGQGNSPLHF         <NA>
211481       FSARRGSEAFF         <NA>
211482       FSARRGTEAFF         <NA>
211483   RSGTRDVGSYNEQFF         <NA>

[305885 rows x 2 columns]
```

[ ]:
```python
mh_gene_symbols = df[["MHC Allele Names", "Chain 1 Species"]].rename(
    columns={"MHC Allele Names": "gene", "Chain 1 Species": "species"}
)
mh_gene_symbols = mh_gene_symbols[mh_gene_symbols["gene"].notna()]
mh_gene_symbols["gene"] = mh_gene_symbols["gene"].str.split(", ")
```

```
mh_gene_symbols = mh_gene_symbols.explode("gene")
mh_gene_symbols["gene"] = mh_gene_symbols["gene"].str.split("/")
mh_gene_symbols = mh_gene_symbols.explode("gene")
mh_gene_symbols = mh_gene_symbols.drop_duplicates()
```

[ ]:
```
mh_gene_symbols
```

[ ]:
```
                  gene      species
145        HLA-E*01:01  homosapiens
145        HLA-E*01:03  homosapiens
147             HLA-B8  homosapiens
149        HLA-B*44:05  homosapiens
150        HLA-B*44:02  homosapiens
…                    …            …
209423        HLA-DR11  homosapiens
209427  HLA-DQB1*05:02  homosapiens
211262  HLA-DPA1*01:03         <NA>
211262     DPB1*04:01          <NA>
211277     DRB3*02:02          <NA>

[284 rows x 2 columns]
```

[ ]:
```
epitopes = df[["Description"]].rename(columns={
    "Description": "epitope"
}).drop_duplicates()
```

[ ]:
```
epitopes
```

[ ]:
```
                             epitope
145                        VMAPRTLIL
147                        FLRGRAYGL
148                        FLRGRFYGL
149                        EEYLQAFTY
154                        EEYLKAWTF
…                                  …
211266                 RSALEDLLFSKVV
211277  PITMQCIKHYVGNGNAINELKFHPR
211303                 RSLLEDLLFNKVK
211305                 RSFVEDLLFDKVV
211390             NLLLQYGSFCTQLNRAL

[1996 rows x 1 columns]
```

1.2 Test TR standardization

[ ]:
```
tr_gene_symbols["corrected"] = tr_gene_symbols.apply(
    lambda row: pd.NA
    if isna(row["gene"])
    else tidytcells.tr.standardise(row["gene"], species="homosapiens" if␣
 ↪isna(row["species"]) else row["species"]),
    axis=1,
)
```

```
/home/yutanagano/Projects/tidytcells/src/tidytcells/_utils/warnings.py:7:
UserWarning: Failed to standardize "12D-2" for species musmusculus: unrecognised
gene name. Attempted fix: "TR12D-2".
  warn(
/home/yutanagano/Projects/tidytcells/src/tidytcells/_utils/warnings.py:7:
UserWarning: Failed to standardize "TCRAV1-4" for species homosapiens:
unrecognised gene name. Attempted fix: "TRAV1-4".
  warn(
/home/yutanagano/Projects/tidytcells/src/tidytcells/_utils/warnings.py:7:
UserWarning: Failed to standardize "TCRAV1-5" for species homosapiens:
unrecognised gene name. Attempted fix: "TRAV1-5".
  warn(
/home/yutanagano/Projects/tidytcells/src/tidytcells/_utils/warnings.py:7:
UserWarning: Failed to standardize "TCRAV2-3" for species homosapiens:
unrecognised gene name. Attempted fix: "TRAV2-3".
  warn(
/home/yutanagano/Projects/tidytcells/src/tidytcells/_utils/warnings.py:7:
UserWarning: Failed to standardize "TCRAV9*02" for species homosapiens:
nonexistent allele for recognised gene. Attempted fix: "TRAV9-1*02".
  warn(
/home/yutanagano/Projects/tidytcells/src/tidytcells/_utils/warnings.py:7:
UserWarning: Failed to standardize "TCRAV2-2" for species homosapiens:
unrecognised gene name. Attempted fix: "TRAV2-2".
  warn(
/home/yutanagano/Projects/tidytcells/src/tidytcells/_utils/warnings.py:7:
UserWarning: Failed to standardize "TCRAV12-4" for species homosapiens:
unrecognised gene name. Attempted fix: "TRAV12-4".
  warn(
/home/yutanagano/Projects/tidytcells/src/tidytcells/_utils/warnings.py:7:
UserWarning: Failed to standardize "TCRAV13/1*02 (F)" for species homosapiens:
unrecognised gene name. Attempted fix: "TRAV13/DV1*02".
  warn(
/home/yutanagano/Projects/tidytcells/src/tidytcells/_utils/warnings.py:7:
UserWarning: Failed to standardize "TCRAV53" for species musmusculus:
unrecognised gene name. Attempted fix: "TRAV53".
  warn(
/home/yutanagano/Projects/tidytcells/src/tidytcells/_utils/warnings.py:7:
UserWarning: Failed to standardize "TCRAV24*0" for species homosapiens:
```

```
nonexistent allele for recognised gene. Attempted fix: "TRAV24*00".
  warn(
/home/yutanagano/Projects/tidytcells/src/tidytcells/_utils/warnings.py:7:
UserWarning: Failed to standardize "mTRDV2-2" for species homosapiens:
unrecognised gene name. Attempted fix: "TRMTRDV2-2".
  warn(
/home/yutanagano/Projects/tidytcells/src/tidytcells/_utils/warnings.py:7:
UserWarning: Failed to standardize "TRBV14DV4" for species homosapiens:
unrecognised gene name. Attempted fix: "TRBV14/DV4".
  warn(
/home/yutanagano/Projects/tidytcells/src/tidytcells/_utils/warnings.py:7:
UserWarning: Failed to standardize "TRBV23VD6" for species homosapiens:
unrecognised gene name. Attempted fix: "TRBV23VD6".
  warn(
/home/yutanagano/Projects/tidytcells/src/tidytcells/_utils/warnings.py:7:
UserWarning: Failed to standardize "TRBV29DV5" for species homosapiens:
unrecognised gene name. Attempted fix: "TRBV29/DV5".
  warn(
/home/yutanagano/Projects/tidytcells/src/tidytcells/_utils/warnings.py:7:
UserWarning: Failed to standardize "TRDAV1*01" for species homosapiens:
unrecognised gene name. Attempted fix: "TRDAV1*01".
  warn(
/home/yutanagano/Projects/tidytcells/src/tidytcells/_utils/warnings.py:7:
UserWarning: Failed to standardize "TCRVJ9" for species homosapiens:
unrecognised gene name. Attempted fix: "TRVJ9".
  warn(
/home/yutanagano/Projects/tidytcells/src/tidytcells/_utils/warnings.py:7:
UserWarning: Failed to standardize "57" for species musmusculus: unrecognised
gene name. Attempted fix: "TR57".
  warn(
/home/yutanagano/Projects/tidytcells/src/tidytcells/_utils/warnings.py:7:
UserWarning: Failed to standardize "TCRAV57*01" for species homosapiens:
unrecognised gene name. Attempted fix: "TRAV57*01".
  warn(
/home/yutanagano/Projects/tidytcells/src/tidytcells/_utils/warnings.py:7:
UserWarning: Failed to standardize "TRAJ37-2" for species homosapiens:
unrecognised gene name. Attempted fix: "TRAJ37-2".
  warn(
/home/yutanagano/Projects/tidytcells/src/tidytcells/_utils/warnings.py:7:
UserWarning: Failed to standardize "TCRAJ1-3" for species homosapiens:
unrecognised gene name. Attempted fix: "TRAJ1-3".
  warn(
/home/yutanagano/Projects/tidytcells/src/tidytcells/_utils/warnings.py:7:
UserWarning: Failed to standardize "TCRAJ17-9" for species homosapiens:
unrecognised gene name. Attempted fix: "TRAJ17-9".
  warn(
/home/yutanagano/Projects/tidytcells/src/tidytcells/_utils/warnings.py:7:
UserWarning: Failed to standardize "TCRAJ16-5" for species homosapiens:
```

```
unrecognised gene name. Attempted fix: "TRAJ16-5".
  warn(
/home/yutanagano/Projects/tidytcells/src/tidytcells/_utils/warnings.py:7:
UserWarning: Failed to standardize "TCRAJ1-8" for species homosapiens:
unrecognised gene name. Attempted fix: "TRAJ1-8".
  warn(
/home/yutanagano/Projects/tidytcells/src/tidytcells/_utils/warnings.py:7:
UserWarning: Failed to standardize "TCRAJ3-2" for species homosapiens:
unrecognised gene name. Attempted fix: "TRAJ3-2".
  warn(
/home/yutanagano/Projects/tidytcells/src/tidytcells/_utils/warnings.py:7:
UserWarning: Failed to standardize "TCRAJ9-14" for species homosapiens:
unrecognised gene name. Attempted fix: "TRAJ9-14".
  warn(
/home/yutanagano/Projects/tidytcells/src/tidytcells/_utils/warnings.py:7:
UserWarning: Failed to standardize "TCRAJ9-7" for species homosapiens:
unrecognised gene name. Attempted fix: "TRAJ9-7".
  warn(
/home/yutanagano/Projects/tidytcells/src/tidytcells/_utils/warnings.py:7:
UserWarning: Failed to standardize "TCRAJ17-5" for species homosapiens:
unrecognised gene name. Attempted fix: "TRAJ17-5".
  warn(
/home/yutanagano/Projects/tidytcells/src/tidytcells/_utils/warnings.py:7:
UserWarning: Failed to standardize "TCRAJ9-11" for species homosapiens:
unrecognised gene name. Attempted fix: "TRAJ9-11".
  warn(
/home/yutanagano/Projects/tidytcells/src/tidytcells/_utils/warnings.py:7:
UserWarning: Failed to standardize "TCRAJ17-11" for species homosapiens:
unrecognised gene name. Attempted fix: "TRAJ17-11".
  warn(
/home/yutanagano/Projects/tidytcells/src/tidytcells/_utils/warnings.py:7:
UserWarning: Failed to standardize "TCRAJ431" for species homosapiens:
unrecognised gene name. Attempted fix: "TRAJ431".
  warn(
/home/yutanagano/Projects/tidytcells/src/tidytcells/_utils/warnings.py:7:
UserWarning: Failed to standardize "TCRAJ151" for species homosapiens:
unrecognised gene name. Attempted fix: "TRAJ151".
  warn(
/home/yutanagano/Projects/tidytcells/src/tidytcells/_utils/warnings.py:7:
UserWarning: Failed to standardize "TCRAJ42-4" for species homosapiens:
unrecognised gene name. Attempted fix: "TRAJ42-4".
  warn(
/home/yutanagano/Projects/tidytcells/src/tidytcells/_utils/warnings.py:7:
UserWarning: Failed to standardize "TCRAJ42-2" for species homosapiens:
unrecognised gene name. Attempted fix: "TRAJ42-2".
  warn(
/home/yutanagano/Projects/tidytcells/src/tidytcells/_utils/warnings.py:7:
UserWarning: Failed to standardize "TRAJA5.1" for species homosapiens:
```

```
unrecognised gene name. Attempted fix: "TRAJA5-1".
  warn(
/home/yutanagano/Projects/tidytcells/src/tidytcells/_utils/warnings.py:7:
UserWarning: Failed to standardize "TRAJ2-7" for species homosapiens:
unrecognised gene name. Attempted fix: "TRAJ2-7".
  warn(
/home/yutanagano/Projects/tidytcells/src/tidytcells/_utils/warnings.py:7:
UserWarning: Failed to standardize "TRAVJ4" for species musmusculus:
unrecognised gene name. Attempted fix: "TRAVJ4".
  warn(
/home/yutanagano/Projects/tidytcells/src/tidytcells/_utils/warnings.py:7:
UserWarning: Failed to standardize "TRAJ10" for species musmusculus:
unrecognised gene name. Attempted fix: "TRAJ10".
  warn(
/home/yutanagano/Projects/tidytcells/src/tidytcells/_utils/warnings.py:7:
UserWarning: Failed to standardize "TRVB13-1*02" for species musmusculus:
unrecognised gene name. Attempted fix: "TRVB13-1*02".
  warn(
/home/yutanagano/Projects/tidytcells/src/tidytcells/_utils/warnings.py:7:
UserWarning: Failed to standardize "1" for species musmusculus: unrecognised
gene name. Attempted fix: "TR1".
  warn(
/home/yutanagano/Projects/tidytcells/src/tidytcells/_utils/warnings.py:7:
UserWarning: Failed to standardize "TRVB13-2*01" for species musmusculus:
unrecognised gene name. Attempted fix: "TRVB13-2*01".
  warn(
/home/yutanagano/Projects/tidytcells/src/tidytcells/_utils/warnings.py:7:
UserWarning: Failed to standardize "TCRBV15-2" for species homosapiens:
unrecognised gene name. Attempted fix: "TRBV15-2".
  warn(
/home/yutanagano/Projects/tidytcells/src/tidytcells/_utils/warnings.py:7:
UserWarning: Failed to standardize "TRBV13-2*01" for species homosapiens:
unrecognised gene name. Attempted fix: "TRBV13-2*01".
  warn(
/home/yutanagano/Projects/tidytcells/src/tidytcells/_utils/warnings.py:7:
UserWarning: Failed to standardize "TCRB6-2" for species homosapiens:
unrecognised gene name. Attempted fix: "TRB6-2".
  warn(
/home/yutanagano/Projects/tidytcells/src/tidytcells/_utils/warnings.py:7:
UserWarning: Failed to standardize "TCRBV13-3" for species homosapiens:
unrecognised gene name. Attempted fix: "TRBV13-3".
  warn(
/home/yutanagano/Projects/tidytcells/src/tidytcells/_utils/warnings.py:7:
UserWarning: Failed to standardize "TCRBV3-1 or TCRBV3-2" for species
homosapiens: unrecognised gene name. Attempted fix: "TRBV3-1/ORTRBV3-2".
  warn(
/home/yutanagano/Projects/tidytcells/src/tidytcells/_utils/warnings.py:7:
UserWarning: Failed to standardize "TCRBV12-3 or TCRBV12-4" for species
```

```
homosapiens: unrecognised gene name. Attempted fix: "TRBV12-3/ORTRBV12-4".
  warn(
/home/yutanagano/Projects/tidytcells/src/tidytcells/_utils/warnings.py:7:
UserWarning: Failed to standardize "TRBV13-2" for species homosapiens:
unrecognised gene name. Attempted fix: "TRBV13-2".
  warn(
/home/yutanagano/Projects/tidytcells/src/tidytcells/_utils/warnings.py:7:
UserWarning: Failed to standardize "TRBV13-3*01" for species homosapiens:
unrecognised gene name. Attempted fix: "TRBV13-3*01".
  warn(
/home/yutanagano/Projects/tidytcells/src/tidytcells/_utils/warnings.py:7:
UserWarning: Failed to standardize "TRBV13-2*01 " for species homosapiens:
unrecognised gene name. Attempted fix: "TRBV13-2*01".
  warn(
/home/yutanagano/Projects/tidytcells/src/tidytcells/_utils/warnings.py:7:
UserWarning: Failed to standardize "TRBV13-3" for species homosapiens:
unrecognised gene name. Attempted fix: "TRBV13-3".
  warn(
/home/yutanagano/Projects/tidytcells/src/tidytcells/_utils/warnings.py:7:
UserWarning: Failed to standardize "TCRBV21-3" for species homosapiens:
unrecognised gene name. Attempted fix: "TRBV21-3".
  warn(
/home/yutanagano/Projects/tidytcells/src/tidytcells/_utils/warnings.py:7:
UserWarning: Failed to standardize "TCRBV13-6" for species homosapiens:
unrecognised gene name. Attempted fix: "TRBV13-6".
  warn(
/home/yutanagano/Projects/tidytcells/src/tidytcells/_utils/warnings.py:7:
UserWarning: Failed to standardize "TCRB17-1" for species homosapiens:
unrecognised gene name. Attempted fix: "TRB17-1".
  warn(
/home/yutanagano/Projects/tidytcells/src/tidytcells/_utils/warnings.py:7:
UserWarning: Failed to standardize "TCRVB6" for species homosapiens:
unrecognised gene name. Attempted fix: "TRVB6".
  warn(
/home/yutanagano/Projects/tidytcells/src/tidytcells/_utils/warnings.py:7:
UserWarning: Failed to standardize "TCRVB06" for species homosapiens:
unrecognised gene name. Attempted fix: "TRVB6".
  warn(
/home/yutanagano/Projects/tidytcells/src/tidytcells/_utils/warnings.py:7:
UserWarning: Failed to standardize "TCRVB12" for species homosapiens:
unrecognised gene name. Attempted fix: "TRVB12".
  warn(
/home/yutanagano/Projects/tidytcells/src/tidytcells/_utils/warnings.py:7:
UserWarning: Failed to standardize "TCRBV8-3" for species homosapiens:
unrecognised gene name. Attempted fix: "TRBV8-3".
  warn(
/home/yutanagano/Projects/tidytcells/src/tidytcells/_utils/warnings.py:7:
UserWarning: Failed to standardize "TCRBV8.3" for species homosapiens:
```

```
unrecognised gene name. Attempted fix: "TRBV8-3".
  warn(
/home/yutanagano/Projects/tidytcells/src/tidytcells/_utils/warnings.py:7:
UserWarning: Failed to standardize "TCRBV31" for species homosapiens:
unrecognised gene name. Attempted fix: "TRBV31".
  warn(
/home/yutanagano/Projects/tidytcells/src/tidytcells/_utils/warnings.py:7:
UserWarning: Failed to standardize "TCRVB8-2" for species homosapiens:
unrecognised gene name. Attempted fix: "TRVB8-2".
  warn(
/home/yutanagano/Projects/tidytcells/src/tidytcells/_utils/warnings.py:7:
UserWarning: Failed to standardize "TCRBV3-3" for species homosapiens:
unrecognised gene name. Attempted fix: "TRBV3-3".
  warn(
/home/yutanagano/Projects/tidytcells/src/tidytcells/_utils/warnings.py:7:
UserWarning: Failed to standardize "TCRBV12-3/4" for species homosapiens:
unrecognised gene name. Attempted fix: "TRBV12-3/4".
  warn(
/home/yutanagano/Projects/tidytcells/src/tidytcells/_utils/warnings.py:7:
UserWarning: Failed to standardize "TCRBB27" for species homosapiens:
unrecognised gene name. Attempted fix: "TRBB27".
  warn(
/home/yutanagano/Projects/tidytcells/src/tidytcells/_utils/warnings.py:7:
UserWarning: Failed to standardize "TRBV13-2*04" for species homosapiens:
unrecognised gene name. Attempted fix: "TRBV13-2*04".
  warn(
/home/yutanagano/Projects/tidytcells/src/tidytcells/_utils/warnings.py:7:
UserWarning: Failed to standardize "2-1 (two-one)" for species musmusculus:
unrecognised gene name. Attempted fix: "TR2-1(TWO-ONE)".
  warn(
/home/yutanagano/Projects/tidytcells/src/tidytcells/_utils/warnings.py:7:
UserWarning: Failed to standardize "TCRJ1-4*01" for species homosapiens:
unrecognised gene name. Attempted fix: "TRJ1-4*01".
  warn(
/home/yutanagano/Projects/tidytcells/src/tidytcells/_utils/warnings.py:7:
UserWarning: Failed to standardize "TCRBJ2-2*02" for species homosapiens:
nonexistent allele for recognised gene. Attempted fix: "TRBJ2-2*02".
  warn(
/home/yutanagano/Projects/tidytcells/src/tidytcells/_utils/warnings.py:7:
UserWarning: Failed to standardize "TCRBJ2*07" for species homosapiens:
nonexistent allele for recognised gene. Attempted fix: "TRBJ2-1*07".
  warn(
/home/yutanagano/Projects/tidytcells/src/tidytcells/_utils/warnings.py:7:
UserWarning: Failed to standardize "TCRBJ1*03" for species homosapiens:
nonexistent allele for recognised gene. Attempted fix: "TRBJ1-1*03".
  warn(
/home/yutanagano/Projects/tidytcells/src/tidytcells/_utils/warnings.py:7:
UserWarning: Failed to standardize "TCRBJ1*02" for species homosapiens:
```

```
nonexistent allele for recognised gene. Attempted fix: "TRBJ1-1*02".
  warn(
/home/yutanagano/Projects/tidytcells/src/tidytcells/_utils/warnings.py:7:
UserWarning: Failed to standardize "TCRBJ2*02" for species homosapiens:
nonexistent allele for recognised gene. Attempted fix: "TRBJ2-1*02".
  warn(
/home/yutanagano/Projects/tidytcells/src/tidytcells/_utils/warnings.py:7:
UserWarning: Failed to standardize "TCRBJ2*03" for species homosapiens:
nonexistent allele for recognised gene. Attempted fix: "TRBJ2-1*03".
  warn(
/home/yutanagano/Projects/tidytcells/src/tidytcells/_utils/warnings.py:7:
UserWarning: Failed to standardize "TCRBJ1*04" for species homosapiens:
nonexistent allele for recognised gene. Attempted fix: "TRBJ1-1*04".
  warn(
/home/yutanagano/Projects/tidytcells/src/tidytcells/_utils/warnings.py:7:
UserWarning: Failed to standardize "TCRBJ2*04" for species homosapiens:
nonexistent allele for recognised gene. Attempted fix: "TRBJ2-1*04".
  warn(
/home/yutanagano/Projects/tidytcells/src/tidytcells/_utils/warnings.py:7:
UserWarning: Failed to standardize "TCRBJ2*05" for species homosapiens:
nonexistent allele for recognised gene. Attempted fix: "TRBJ2-1*05".
  warn(
/home/yutanagano/Projects/tidytcells/src/tidytcells/_utils/warnings.py:7:
UserWarning: Failed to standardize "TRBJ1-4*02" for species homosapiens:
nonexistent allele for recognised gene. Attempted fix: "TRBJ1-4*02".
  warn(
/home/yutanagano/Projects/tidytcells/src/tidytcells/_utils/warnings.py:7:
UserWarning: Failed to standardize "TRBJ3-2" for species homosapiens:
unrecognised gene name. Attempted fix: "TRBJ3-2".
  warn(
/home/yutanagano/Projects/tidytcells/src/tidytcells/_utils/warnings.py:7:
UserWarning: Failed to standardize "TRAV14D-3/DV8*02" for species homosapiens:
unrecognised gene name. Attempted fix: "TRAV14D-3/DV8*02".
  warn(
/home/yutanagano/Projects/tidytcells/src/tidytcells/_utils/warnings.py:14:
UserWarning: Unsupported species: "syntheticconstruct". Skipping TR
standardisation.
  warn(f'Unsupported species: "{species}". ' f"Skipping {gene_type}
standardisation.")
/home/yutanagano/Projects/tidytcells/src/tidytcells/_utils/warnings.py:14:
UserWarning: Unsupported species: "macacamulatta". Skipping TR standardisation.
  warn(f'Unsupported species: "{species}". ' f"Skipping {gene_type}
standardisation.")
/home/yutanagano/Projects/tidytcells/src/tidytcells/_utils/warnings.py:14:
UserWarning: Unsupported species: "gallusgallus". Skipping TR standardisation.
  warn(f'Unsupported species: "{species}". ' f"Skipping {gene_type}
standardisation.")
```

[ ]:
```
num_tr_successes = (
    tr_gene_symbols["corrected"].notna() & (
        tr_gene_symbols["species"].isna() |
        tr_gene_symbols["species"].map(lambda x: type(x) == str and x in␣
↪("homosapiens", "musmusculus"))
    )
).sum()
num_tr_total = len(tr_gene_symbols)

where_tr_changed = tr_gene_symbols[tr_gene_symbols["gene"] !=␣
↪tr_gene_symbols["corrected"]]
num_successful_tr_changes = where_tr_changed["corrected"].notna().sum()
num_tr_changes = len(where_tr_changed)

print(f"total number of IMGT-compliant TR symbols:          {num_tr_successes}")
print(f"original number of unique TR symbols:               {num_tr_total}")
print(f"fraction of TR symbols standardized:                {num_tr_successes /␣
↪num_tr_total}\n")

print(f"number of TR symbols originally IMGT-noncompliant:  {num_tr_changes}")
print(f"number of IMGT-noncompliant symbols fixed:         ␣
↪{num_successful_tr_changes}")
print(f"fraction of IMGT-noncompliant symbols standardized:␣
↪{num_successful_tr_changes / num_tr_changes}")
```

```
total number of IMGT-compliant TR symbols:          2127
original number of unique TR symbols:               2225
fraction of TR symbols standardized:                0.9559550561797753

number of TR symbols originally IMGT-noncompliant:  1217
number of IMGT-noncompliant symbols fixed:          1137
fraction of IMGT-noncompliant symbols standardized: 0.934264585045193
```

[ ]:
```
tr_gene_symbols.to_csv("tr_gene_symbols.csv", index=False)
```

1.3 Test MH standardization

[ ]:
```
mh_gene_symbols["corrected"] = mh_gene_symbols.apply(
    lambda row: pd.NA
    if pd.isna(row["gene"])
    else tidytcells.mh.standardise(row["gene"], species="homosapiens" if␣
↪isna(row["species"]) else row["species"]),
    axis=1,
)
```

```
/home/yutanagano/Projects/tidytcells/src/tidytcells/_utils/warnings.py:7:
UserWarning: Failed to standardize "E63Q mutant" for species homosapiens:
unrecognised gene name. Attempted fix: "HLA-E63QMUTANT".
```

```
  warn(
/home/yutanagano/Projects/tidytcells/src/tidytcells/_utils/warnings.py:7:
UserWarning: Failed to standardize "human CD1d" for species homosapiens:
unrecognised gene name. Attempted fix: "HLA-HUMANCD1D".
  warn(
/home/yutanagano/Projects/tidytcells/src/tidytcells/_utils/warnings.py:7:
UserWarning: Failed to standardize "human MR1" for species homosapiens:
unrecognised gene name. Attempted fix: "HLA-HUMANMR*1".
  warn(
/home/yutanagano/Projects/tidytcells/src/tidytcells/_utils/warnings.py:7:
UserWarning: Failed to standardize "H2-IAu" for species musmusculus:
unrecognised gene name. Attempted fix: "H2-IAU".
  warn(
/home/yutanagano/Projects/tidytcells/src/tidytcells/_utils/warnings.py:7:
UserWarning: Failed to standardize "H2-IAb" for species musmusculus:
unrecognised gene name. Attempted fix: "H2-IAB".
  warn(
/home/yutanagano/Projects/tidytcells/src/tidytcells/_utils/warnings.py:7:
UserWarning: Failed to standardize "H2-IAg7" for species musmusculus:
unrecognised gene name. Attempted fix: "H2-IAG7".
  warn(
/home/yutanagano/Projects/tidytcells/src/tidytcells/_utils/warnings.py:7:
UserWarning: Failed to standardize "H2-Ld" for species musmusculus: unrecognised
gene name. Attempted fix: "H2-LD".
  warn(
/home/yutanagano/Projects/tidytcells/src/tidytcells/_utils/warnings.py:7:
UserWarning: Failed to standardize "T69A" for species homosapiens: unrecognised
gene name. Attempted fix: "HLA-T69A".
  warn(
/home/yutanagano/Projects/tidytcells/src/tidytcells/_utils/warnings.py:7:
UserWarning: Failed to standardize "Q155A mutant" for species homosapiens:
unrecognised gene name. Attempted fix: "HLA-Q155AMUTANT".
  warn(
/home/yutanagano/Projects/tidytcells/src/tidytcells/_utils/warnings.py:7:
UserWarning: Failed to standardize "H2-Kb Y22F" for species musmusculus:
unrecognised gene name. Attempted fix: "H2-KBY22F".
  warn(
/home/yutanagano/Projects/tidytcells/src/tidytcells/_utils/warnings.py:7:
UserWarning: Failed to standardize "M23I" for species musmusculus: unrecognised
gene name. Attempted fix: "M23I".
  warn(
/home/yutanagano/Projects/tidytcells/src/tidytcells/_utils/warnings.py:7:
UserWarning: Failed to standardize "E24S" for species musmusculus: unrecognised
gene name. Attempted fix: "E24S".
  warn(
/home/yutanagano/Projects/tidytcells/src/tidytcells/_utils/warnings.py:7:
UserWarning: Failed to standardize "D30N mutant" for species musmusculus:
unrecognised gene name. Attempted fix: "D30NMUTANT".
```

```
  warn(
/home/yutanagano/Projects/tidytcells/src/tidytcells/_utils/warnings.py:7:
UserWarning: Failed to standardize "H2-Kb" for species musmusculus: unrecognised
gene name. Attempted fix: "H2-KB".
  warn(
/home/yutanagano/Projects/tidytcells/src/tidytcells/_utils/warnings.py:7:
UserWarning: Failed to standardize "HLA-A*02:01" for species musmusculus:
unrecognised gene name. Attempted fix: "HLA-A*02".
  warn(
/home/yutanagano/Projects/tidytcells/src/tidytcells/_utils/warnings.py:7:
UserWarning: Failed to standardize "HLA-A*02:01 W167A mutant" for species
musmusculus: unrecognised gene name. Attempted fix: "HLA-A*02".
  warn(
/home/yutanagano/Projects/tidytcells/src/tidytcells/_utils/warnings.py:7:
UserWarning: Failed to standardize "HLA-A*02:01 T163A mutant" for species
musmusculus: unrecognised gene name. Attempted fix: "HLA-A*02".
  warn(
/home/yutanagano/Projects/tidytcells/src/tidytcells/_utils/warnings.py:7:
UserWarning: Failed to standardize "HLA-A*02:01 K66A mutant" for species
musmusculus: unrecognised gene name. Attempted fix: "HLA-A*02".
  warn(
/home/yutanagano/Projects/tidytcells/src/tidytcells/_utils/warnings.py:7:
UserWarning: Failed to standardize "HLA-A*02:01 Q155A mutant" for species
musmusculus: unrecognised gene name. Attempted fix: "HLA-A*02".
  warn(
/home/yutanagano/Projects/tidytcells/src/tidytcells/_utils/warnings.py:7:
UserWarning: Failed to standardize "human CD1d" for species musmusculus:
unrecognised gene name. Attempted fix: "HUMANCD1D".
  warn(
/home/yutanagano/Projects/tidytcells/src/tidytcells/_utils/warnings.py:7:
UserWarning: Failed to standardize "mouse CD1d" for species musmusculus:
unrecognised gene name. Attempted fix: "MOUSECD1D".
  warn(
/home/yutanagano/Projects/tidytcells/src/tidytcells/_utils/warnings.py:7:
UserWarning: Failed to standardize "mouse CD1d" for species homosapiens:
unrecognised gene name. Attempted fix: "HLA-MOUSECD1D".
  warn(
/home/yutanagano/Projects/tidytcells/src/tidytcells/_utils/warnings.py:7:
UserWarning: Failed to standardize "HLA-DQ1" for species homosapiens:
unrecognised gene name. Attempted fix: "HLA-DQ*1".
  warn(
/home/yutanagano/Projects/tidytcells/src/tidytcells/_utils/warnings.py:7:
UserWarning: Failed to standardize "H2-IEk" for species musmusculus:
unrecognised gene name. Attempted fix: "H2-IEK".
  warn(
/home/yutanagano/Projects/tidytcells/src/tidytcells/_utils/warnings.py:7:
UserWarning: Failed to standardize "HLA-DR" for species homosapiens:
unrecognised gene name. Attempted fix: "HLA-DR".
```

```
  warn(
/home/yutanagano/Projects/tidytcells/src/tidytcells/_utils/warnings.py:7:
UserWarning: Failed to standardize "human CD1b" for species homosapiens:
unrecognised gene name. Attempted fix: "HLA-HUMANCD1B".
  warn(
/home/yutanagano/Projects/tidytcells/src/tidytcells/_utils/warnings.py:7:
UserWarning: Failed to standardize "human MR1 K43A mutant" for species
homosapiens: unrecognised gene name. Attempted fix: "HLA-HUMANMR1K43AMUTANT".
  warn(
/home/yutanagano/Projects/tidytcells/src/tidytcells/_utils/warnings.py:7:
UserWarning: Failed to standardize "cattle MR1" for species homosapiens:
unrecognised gene name. Attempted fix: "HLA-CATTLEMR*1".
  warn(
/home/yutanagano/Projects/tidytcells/src/tidytcells/_utils/warnings.py:7:
UserWarning: Failed to standardize "human CD1a" for species homosapiens:
unrecognised gene name. Attempted fix: "HLA-HUMANCD1A".
  warn(
/home/yutanagano/Projects/tidytcells/src/tidytcells/_utils/warnings.py:7:
UserWarning: Failed to standardize "mouse MR1" for species homosapiens:
unrecognised gene name. Attempted fix: "HLA-MOUSEMR*1".
  warn(
/home/yutanagano/Projects/tidytcells/src/tidytcells/_utils/warnings.py:7:
UserWarning: Failed to standardize "human CD1c" for species homosapiens:
unrecognised gene name. Attempted fix: "HLA-HUMANCD1C".
  warn(
/home/yutanagano/Projects/tidytcells/src/tidytcells/_utils/warnings.py:7:
UserWarning: Failed to standardize "HLA class II" for species homosapiens:
unrecognised gene name. Attempted fix: "HLA-HLACLASSII".
  warn(
/home/yutanagano/Projects/tidytcells/src/tidytcells/_utils/warnings.py:7:
UserWarning: Failed to standardize "H2-IAk" for species musmusculus:
unrecognised gene name. Attempted fix: "H2-IAK".
  warn(
/home/yutanagano/Projects/tidytcells/src/tidytcells/_utils/warnings.py:7:
UserWarning: Failed to standardize "H2 class II" for species musmusculus:
unrecognised gene name. Attempted fix: "H2CLASSII".
  warn(
/home/yutanagano/Projects/tidytcells/src/tidytcells/_utils/warnings.py:7:
UserWarning: Failed to standardize "H2-s class I" for species musmusculus:
unrecognised gene name. Attempted fix: "H2-SCLASSI".
  warn(
/home/yutanagano/Projects/tidytcells/src/tidytcells/_utils/warnings.py:7:
UserWarning: Failed to standardize "H2-IAs" for species musmusculus:
unrecognised gene name. Attempted fix: "H2-IAS".
  warn(
/home/yutanagano/Projects/tidytcells/src/tidytcells/_utils/warnings.py:7:
UserWarning: Failed to standardize "H2-b class I" for species musmusculus:
unrecognised gene name. Attempted fix: "H2-BCLASSI".
```

```
  warn(
/home/yutanagano/Projects/tidytcells/src/tidytcells/_utils/warnings.py:7:
UserWarning: Failed to standardize "H2-b class II" for species musmusculus:
unrecognised gene name. Attempted fix: "H2-BCLASSII".
  warn(
/home/yutanagano/Projects/tidytcells/src/tidytcells/_utils/warnings.py:7:
UserWarning: Failed to standardize "H2 class I" for species musmusculus:
unrecognised gene name. Attempted fix: "H2CLASSI".
  warn(
/home/yutanagano/Projects/tidytcells/src/tidytcells/_utils/warnings.py:7:
UserWarning: Failed to standardize "H2-u class II" for species musmusculus:
unrecognised gene name. Attempted fix: "H2-UCLASSII".
  warn(
/home/yutanagano/Projects/tidytcells/src/tidytcells/_utils/warnings.py:7:
UserWarning: Failed to standardize "H2-IAk" for species homosapiens:
unrecognised gene name. Attempted fix: "HLA-H2-IAK".
  warn(
/home/yutanagano/Projects/tidytcells/src/tidytcells/_utils/warnings.py:7:
UserWarning: Failed to standardize "HLA-DR2" for species homosapiens:
unrecognised gene name. Attempted fix: "HLA-DR*2".
  warn(
/home/yutanagano/Projects/tidytcells/src/tidytcells/_utils/warnings.py:7:
UserWarning: Failed to standardize "H2 class II" for species homosapiens:
unrecognised gene name. Attempted fix: "HLA-H2CLASSII".
  warn(
/home/yutanagano/Projects/tidytcells/src/tidytcells/_utils/warnings.py:7:
UserWarning: Failed to standardize "H2-IAd" for species musmusculus:
unrecognised gene name. Attempted fix: "H2-IAD".
  warn(
/home/yutanagano/Projects/tidytcells/src/tidytcells/_utils/warnings.py:7:
UserWarning: Failed to standardize "H2-d class II" for species musmusculus:
unrecognised gene name. Attempted fix: "H2-DCLASSII".
  warn(
/home/yutanagano/Projects/tidytcells/src/tidytcells/_utils/warnings.py:7:
UserWarning: Failed to standardize "H2-IAq" for species musmusculus:
unrecognised gene name. Attempted fix: "H2-IAQ".
  warn(
/home/yutanagano/Projects/tidytcells/src/tidytcells/_utils/warnings.py:7:
UserWarning: Failed to standardize "H2-Db Y159F mutant" for species musmusculus:
unrecognised gene name. Attempted fix: "H2-DBY159FMUTANT".
  warn(
/home/yutanagano/Projects/tidytcells/src/tidytcells/_utils/warnings.py:7:
UserWarning: Failed to standardize "H2-Kd" for species musmusculus: unrecognised
gene name. Attempted fix: "H2-KD".
  warn(
/home/yutanagano/Projects/tidytcells/src/tidytcells/_utils/warnings.py:7:
UserWarning: Failed to standardize "H2-a class II" for species musmusculus:
unrecognised gene name. Attempted fix: "H2-ACLASSII".
```

```
  warn(
/home/yutanagano/Projects/tidytcells/src/tidytcells/_utils/warnings.py:7:
UserWarning: Failed to standardize "Class II" for species musmusculus:
unrecognised gene name. Attempted fix: "CLASSII".
  warn(
/home/yutanagano/Projects/tidytcells/src/tidytcells/_utils/warnings.py:7:
UserWarning: Failed to standardize "allele undetermined" for species
musmusculus: unrecognised gene name. Attempted fix: "ALLELEUNDETERMINED".
  warn(
/home/yutanagano/Projects/tidytcells/src/tidytcells/_utils/warnings.py:7:
UserWarning: Failed to standardize "H2-IAb" for species homosapiens:
unrecognised gene name. Attempted fix: "HLA-H2-IAB".
  warn(
/home/yutanagano/Projects/tidytcells/src/tidytcells/_utils/warnings.py:7:
UserWarning: Failed to standardize "H2-Kb D77S" for species musmusculus:
unrecognised gene name. Attempted fix: "H2-KBD77S".
  warn(
/home/yutanagano/Projects/tidytcells/src/tidytcells/_utils/warnings.py:7:
UserWarning: Failed to standardize "K89A mutant" for species musmusculus:
unrecognised gene name. Attempted fix: "K89AMUTANT".
  warn(
/home/yutanagano/Projects/tidytcells/src/tidytcells/_utils/warnings.py:7:
UserWarning: Failed to standardize "H2-Kb Y22F" for species homosapiens:
unrecognised gene name. Attempted fix: "HLA-H2-KBY22F".
  warn(
/home/yutanagano/Projects/tidytcells/src/tidytcells/_utils/warnings.py:7:
UserWarning: Failed to standardize "M23I" for species homosapiens: unrecognised
gene name. Attempted fix: "HLA-M23I".
  warn(
/home/yutanagano/Projects/tidytcells/src/tidytcells/_utils/warnings.py:7:
UserWarning: Failed to standardize "E24S" for species homosapiens: unrecognised
gene name. Attempted fix: "HLA-E24S".
  warn(
/home/yutanagano/Projects/tidytcells/src/tidytcells/_utils/warnings.py:7:
UserWarning: Failed to standardize "D30N mutant" for species homosapiens:
unrecognised gene name. Attempted fix: "HLA-D30NMUTANT".
  warn(
/home/yutanagano/Projects/tidytcells/src/tidytcells/_utils/warnings.py:7:
UserWarning: Failed to standardize "H2-Kb" for species homosapiens: unrecognised
gene name. Attempted fix: "HLA-H2-KB".
  warn(
/home/yutanagano/Projects/tidytcells/src/tidytcells/_utils/warnings.py:7:
UserWarning: Failed to standardize "HLA-DR3" for species homosapiens:
unrecognised gene name. Attempted fix: "HLA-DR*3".
  warn(
/home/yutanagano/Projects/tidytcells/src/tidytcells/_utils/warnings.py:7:
UserWarning: Failed to standardize "H2-d class II" for species homosapiens:
unrecognised gene name. Attempted fix: "HLA-H2-DCLASSII".
```

```
  warn(
/home/yutanagano/Projects/tidytcells/src/tidytcells/_utils/warnings.py:7:
UserWarning: Failed to standardize "H2-IEd" for species homosapiens:
unrecognised gene name. Attempted fix: "HLA-H2-IED".
  warn(
/home/yutanagano/Projects/tidytcells/src/tidytcells/_utils/warnings.py:7:
UserWarning: Failed to standardize "H2-Db" for species homosapiens: unrecognised
gene name. Attempted fix: "HLA-H2-DB".
  warn(
/home/yutanagano/Projects/tidytcells/src/tidytcells/_utils/warnings.py:7:
UserWarning: Failed to standardize "H2-Kb Y84A mutant" for species homosapiens:
unrecognised gene name. Attempted fix: "HLA-H2-KBY84AMUTANT".
  warn(
/home/yutanagano/Projects/tidytcells/src/tidytcells/_utils/warnings.py:7:
UserWarning: Failed to standardize "H2-Kb Y84C mutant" for species homosapiens:
unrecognised gene name. Attempted fix: "HLA-H2-KBY84CMUTANT".
  warn(
/home/yutanagano/Projects/tidytcells/src/tidytcells/_utils/warnings.py:7:
UserWarning: Failed to standardize "H2 class I" for species homosapiens:
unrecognised gene name. Attempted fix: "HLA-H2CLASSI".
  warn(
/home/yutanagano/Projects/tidytcells/src/tidytcells/_utils/warnings.py:7:
UserWarning: Failed to standardize "H2-b class I" for species homosapiens:
unrecognised gene name. Attempted fix: "HLA-H2-BCLASSI".
  warn(
/home/yutanagano/Projects/tidytcells/src/tidytcells/_utils/warnings.py:7:
UserWarning: Failed to standardize "H2-b class II" for species homosapiens:
unrecognised gene name. Attempted fix: "HLA-H2-BCLASSII".
  warn(
/home/yutanagano/Projects/tidytcells/src/tidytcells/_utils/warnings.py:7:
UserWarning: Failed to standardize "H2-IAg7" for species homosapiens:
unrecognised gene name. Attempted fix: "HLA-H2-IAG7".
  warn(
/home/yutanagano/Projects/tidytcells/src/tidytcells/_utils/warnings.py:7:
UserWarning: Failed to standardize "HLA class I" for species homosapiens:
unrecognised gene name. Attempted fix: "HLA-HLACLASSI".
  warn(
/home/yutanagano/Projects/tidytcells/src/tidytcells/_utils/warnings.py:7:
UserWarning: Failed to standardize "H2-IAs" for species homosapiens:
unrecognised gene name. Attempted fix: "HLA-H2-IAS".
  warn(
/home/yutanagano/Projects/tidytcells/src/tidytcells/_utils/warnings.py:7:
UserWarning: Failed to standardize "H2-IEk" for species homosapiens:
unrecognised gene name. Attempted fix: "HLA-H2-IEK".
  warn(
/home/yutanagano/Projects/tidytcells/src/tidytcells/_utils/warnings.py:14:
UserWarning: Unsupported species: "syntheticconstruct". Skipping MH
standardisation.
```

```
  warn(f'Unsupported species: "{species}". ' f"Skipping {gene_type}
standardisation.")
/home/yutanagano/Projects/tidytcells/src/tidytcells/_utils/warnings.py:7:
UserWarning: Failed to standardize "H2-Kd" for species homosapiens: unrecognised
gene name. Attempted fix: "HLA-H2-KD".
  warn(
/home/yutanagano/Projects/tidytcells/src/tidytcells/_utils/warnings.py:14:
UserWarning: Unsupported species: "unculturedmarineeubacteriumhstpl64". Skipping
MH standardisation.
  warn(f'Unsupported species: "{species}". ' f"Skipping {gene_type}
standardisation.")
/home/yutanagano/Projects/tidytcells/src/tidytcells/_utils/warnings.py:7:
UserWarning: Failed to standardize "HLA-DR1" for species homosapiens:
unrecognised gene name. Attempted fix: "HLA-DR*1".
  warn(
/home/yutanagano/Projects/tidytcells/src/tidytcells/_utils/warnings.py:7:
UserWarning: Failed to standardize "HLA-DQ2" for species homosapiens:
unrecognised gene name. Attempted fix: "HLA-DQ*2".
  warn(
/home/yutanagano/Projects/tidytcells/src/tidytcells/_utils/warnings.py:7:
UserWarning: Failed to standardize "HLA-DP" for species homosapiens:
unrecognised gene name. Attempted fix: "HLA-DP".
  warn(
/home/yutanagano/Projects/tidytcells/src/tidytcells/_utils/warnings.py:7:
UserWarning: Failed to standardize "HLA-DQ" for species homosapiens:
unrecognised gene name. Attempted fix: "HLA-DQ".
  warn(
/home/yutanagano/Projects/tidytcells/src/tidytcells/_utils/warnings.py:7:
UserWarning: Failed to standardize "mouse MR1" for species musmusculus:
unrecognised gene name. Attempted fix: "MOUSEMR1".
  warn(
/home/yutanagano/Projects/tidytcells/src/tidytcells/_utils/warnings.py:7:
UserWarning: Failed to standardize "H2-IAd" for species homosapiens:
unrecognised gene name. Attempted fix: "HLA-H2-IAD".
  warn(
/home/yutanagano/Projects/tidytcells/src/tidytcells/_utils/warnings.py:14:
UserWarning: Unsupported species: "macacamulatta". Skipping MH standardisation.
  warn(f'Unsupported species: "{species}". ' f"Skipping {gene_type}
standardisation.")
/home/yutanagano/Projects/tidytcells/src/tidytcells/_utils/warnings.py:7:
UserWarning: Failed to standardize "H2-IAb I67F" for species musmusculus:
unrecognised gene name. Attempted fix: "H2-IABI67F".
  warn(
/home/yutanagano/Projects/tidytcells/src/tidytcells/_utils/warnings.py:7:
UserWarning: Failed to standardize "R70Q" for species musmusculus: unrecognised
gene name. Attempted fix: "R70Q".
  warn(
/home/yutanagano/Projects/tidytcells/src/tidytcells/_utils/warnings.py:7:
```

```
UserWarning: Failed to standardize "T71K mutant" for species musmusculus:
unrecognised gene name. Attempted fix: "T71KMUTANT".
  warn(
/home/yutanagano/Projects/tidytcells/src/tidytcells/_utils/warnings.py:7:
UserWarning: Failed to standardize "HLA-DR4" for species homosapiens:
unrecognised gene name. Attempted fix: "HLA-DR*4".
  warn(
/home/yutanagano/Projects/tidytcells/src/tidytcells/_utils/warnings.py:7:
UserWarning: Failed to standardize "HLA-A*11:01" for species musmusculus:
unrecognised gene name. Attempted fix: "HLA-A*11".
  warn(
/home/yutanagano/Projects/tidytcells/src/tidytcells/_utils/warnings.py:14:
UserWarning: Unsupported species: "gallusgallus". Skipping MH standardisation.
  warn(f'Unsupported species: "{species}". ' f"Skipping {gene_type}
standardisation.")
/home/yutanagano/Projects/tidytcells/src/tidytcells/_utils/warnings.py:7:
UserWarning: Failed to standardize "HLA-DQ6" for species homosapiens:
unrecognised gene name. Attempted fix: "HLA-DQ*6".
  warn(
/home/yutanagano/Projects/tidytcells/src/tidytcells/_utils/warnings.py:7:
UserWarning: Failed to standardize "HLA-DR7" for species homosapiens:
unrecognised gene name. Attempted fix: "HLA-DR*7".
  warn(
/home/yutanagano/Projects/tidytcells/src/tidytcells/_utils/warnings.py:7:
UserWarning: Failed to standardize "HLA-DRB1*04:01" for species musmusculus:
unrecognised gene name. Attempted fix: "HLA-DRB1*04".
  warn(
/home/yutanagano/Projects/tidytcells/src/tidytcells/_utils/warnings.py:7:
UserWarning: Failed to standardize "HLA-DRB1*04:05" for species musmusculus:
unrecognised gene name. Attempted fix: "HLA-DRB1*04".
  warn(
/home/yutanagano/Projects/tidytcells/src/tidytcells/_utils/warnings.py:7:
UserWarning: Failed to standardize "mouse CD1d1" for species musmusculus:
unrecognised gene name. Attempted fix: "MOUSECD1D1".
  warn(
/home/yutanagano/Projects/tidytcells/src/tidytcells/_utils/warnings.py:7:
UserWarning: Failed to standardize "HLA-DPw2" for species homosapiens:
unrecognised gene name. Attempted fix: "HLA-DPW*2".
  warn(
/home/yutanagano/Projects/tidytcells/src/tidytcells/_utils/warnings.py:7:
UserWarning: Failed to standardize "HLA-DRB1*01:01" for species musmusculus:
unrecognised gene name. Attempted fix: "HLA-DRB1*01".
  warn(
/home/yutanagano/Projects/tidytcells/src/tidytcells/_utils/warnings.py:7:
UserWarning: Failed to standardize "HLA-DR11" for species homosapiens:
unrecognised gene name. Attempted fix: "HLA-DR*11".
  warn(
```

```
[ ]: num_mh_successes = (
         mh_gene_symbols["corrected"].notna() & (
             mh_gene_symbols["species"].isna() |
             mh_gene_symbols["species"].map(lambda x: type(x) == str and x in␣
      ↪("homosapiens", "musmusculus"))
         )
     ).sum()
     num_mh_total = len(mh_gene_symbols)

     where_mh_changed = mh_gene_symbols[mh_gene_symbols["gene"] !=␣
      ↪mh_gene_symbols["corrected"]]
     num_successful_mh_changes = where_mh_changed["corrected"].notna().sum()
     num_mh_changes = len(where_mh_changed)

     print(f"total number of IMGT-compliant MH symbols:          {num_mh_successes}")
     print(f"original number of unique MH symbols:               {num_mh_total}")
     print(f"fraction of MH symbols standardized:                {num_mh_successes /␣
      ↪num_mh_total}\n")

     print(f"number of MH symbols originally IMGT-noncompliant:  {num_mh_changes}")
     print(f"number of IMGT-noncompliant symbols fixed:         ␣
      ↪{num_successful_mh_changes}")
     print(f"fraction of IMGT-noncompliant symbols standardized:␣
      ↪{num_successful_mh_changes / num_mh_changes}")
```

```
total number of IMGT-compliant MH symbols:          173
original number of unique MH symbols:               284
fraction of MH symbols standardized:                0.6091549295774648

number of MH symbols originally IMGT-noncompliant:  151
number of IMGT-noncompliant symbols fixed:          45
fraction of IMGT-noncompliant symbols standardized: 0.2980132450331126
```

```
[ ]: mh_gene_symbols.to_csv("mh_gene_symbols.csv", index=False)
```

1.4 Test junction standardization

```
[ ]: junctions["corrected"] = junctions["junction"].map(tidytcells.junction.
      ↪standardize)
```

```
/home/yutanagano/Projects/tidytcells/src/tidytcells/aa/_standardize.py:80:
UserWarning: Failed to standardize IVRVSHN*G#RDNYGQNFV: not a valid amino acid
sequence.
  warnings.warn(
/home/yutanagano/Projects/tidytcells/src/tidytcells/aa/_standardize.py:80:
UserWarning: Failed to standardize AFTSGTYXYI: not a valid amino acid sequence.
  warnings.warn(
/home/yutanagano/Projects/tidytcells/src/tidytcells/aa/_standardize.py:80:
```

```
UserWarning: Failed to standardize AG4DSQSARQLT: not a valid amino acid
sequence.
  warnings.warn(
/home/yutanagano/Projects/tidytcells/src/tidytcells/aa/_standardize.py:80:
UserWarning: Failed to standardize AGAGSOGNLI: not a valid amino acid sequence.
  warnings.warn(
/home/yutanagano/Projects/tidytcells/src/tidytcells/aa/_standardize.py:80:
UserWarning: Failed to standardize AGAOGSOGNLIF: not a valid amino acid
sequence.
  warnings.warn(
/home/yutanagano/Projects/tidytcells/src/tidytcells/aa/_standardize.py:80:
UserWarning: Failed to standardize AGAVGSSNTGKU: not a valid amino acid
sequence.
  warnings.warn(
/home/yutanagano/Projects/tidytcells/src/tidytcells/aa/_standardize.py:80:
UserWarning: Failed to standardize AGGGSOGNLI: not a valid amino acid sequence.
  warnings.warn(
/home/yutanagano/Projects/tidytcells/src/tidytcells/aa/_standardize.py:80:
UserWarning: Failed to standardize AGGGSQGNRJ: not a valid amino acid sequence.
  warnings.warn(
/home/yutanagano/Projects/tidytcells/src/tidytcells/aa/_standardize.py:80:
UserWarning: Failed to standardize AGRGSOGNLI: not a valid amino acid sequence.
  warnings.warn(
/home/yutanagano/Projects/tidytcells/src/tidytcells/aa/_standardize.py:80:
UserWarning: Failed to standardize AGROVEYGNKLV: not a valid amino acid
sequence.
  warnings.warn(
/home/yutanagano/Projects/tidytcells/src/tidytcells/aa/_standardize.py:80:
UserWarning: Failed to standardize AIIEAROOXII: not a valid amino acid sequence.
  warnings.warn(
/home/yutanagano/Projects/tidytcells/src/tidytcells/aa/_standardize.py:80:
UserWarning: Failed to standardize AMNQAGTAU: not a valid amino acid sequence.
  warnings.warn(
/home/yutanagano/Projects/tidytcells/src/tidytcells/aa/_standardize.py:80:
UserWarning: Failed to standardize AMSGOGGSQGNLI: not a valid amino acid
sequence.
  warnings.warn(
/home/yutanagano/Projects/tidytcells/src/tidytcells/aa/_standardize.py:80:
UserWarning: Failed to standardize APRQGSAROLT: not a valid amino acid sequence.
  warnings.warn(
/home/yutanagano/Projects/tidytcells/src/tidytcells/aa/_standardize.py:80:
UserWarning: Failed to standardize ARSGDGGSQINIJ: not a valid amino acid
sequence.
  warnings.warn(
/home/yutanagano/Projects/tidytcells/src/tidytcells/aa/_standardize.py:80:
UserWarning: Failed to standardize AVEGPGSGAGSYQIJ: not a valid amino acid
sequence.
  warnings.warn(
```

```
/home/yutanagano/Projects/tidytcells/src/tidytcells/aa/_standardize.py:80:
UserWarning: Failed to standardize AVGOSFQKLV: not a valid amino acid sequence.
  warnings.warn(
/home/yutanagano/Projects/tidytcells/src/tidytcells/aa/_standardize.py:80:
UserWarning: Failed to standardize AVNRSNFGNEXUF: not a valid amino acid
sequence.
  warnings.warn(
/home/yutanagano/Projects/tidytcells/src/tidytcells/aa/_standardize.py:80:
UserWarning: Failed to standardize AVNSVNRDDKUF: not a valid amino acid
sequence.
  warnings.warn(
/home/yutanagano/Projects/tidytcells/src/tidytcells/aa/_standardize.py:80:
UserWarning: Failed to standardize AVRDOKII: not a valid amino acid sequence.
  warnings.warn(
/home/yutanagano/Projects/tidytcells/src/tidytcells/aa/_standardize.py:80:
UserWarning: Failed to standardize AVSGGGGSQGNUF: not a valid amino acid
sequence.
  warnings.warn(
/home/yutanagano/Projects/tidytcells/src/tidytcells/aa/_standardize.py:80:
UserWarning: Failed to standardize AVSRVWTA(I: not a valid amino acid sequence.
  warnings.warn(
/home/yutanagano/Projects/tidytcells/src/tidytcells/aa/_standardize.py:80:
UserWarning: Failed to standardize AVSSKNOMR: not a valid amino acid sequence.
  warnings.warn(
/home/yutanagano/Projects/tidytcells/src/tidytcells/aa/_standardize.py:80:
UserWarning: Failed to standardize AVSTGGGJMQJ: not a valid amino acid sequence.
  warnings.warn(
/home/yutanagano/Projects/tidytcells/src/tidytcells/aa/_standardize.py:80:
UserWarning: Failed to standardize AVTKEIGNEXLT: not a valid amino acid
sequence.
  warnings.warn(
/home/yutanagano/Projects/tidytcells/src/tidytcells/aa/_standardize.py:80:
UserWarning: Failed to standardize AVXGNEKLT: not a valid amino acid sequence.
  warnings.warn(
/home/yutanagano/Projects/tidytcells/src/tidytcells/aa/_standardize.py:80:
UserWarning: Failed to standardize AVXKGSGGYQKVT: not a valid amino acid
sequence.
  warnings.warn(
/home/yutanagano/Projects/tidytcells/src/tidytcells/aa/_standardize.py:80:
UserWarning: Failed to standardize VDNQGGXLI: not a valid amino acid sequence.
  warnings.warn(
/home/yutanagano/Projects/tidytcells/src/tidytcells/aa/_standardize.py:80:
UserWarning: Failed to standardize VGOSSGTYKYI: not a valid amino acid sequence.
  warnings.warn(
/home/yutanagano/Projects/tidytcells/src/tidytcells/aa/_standardize.py:80:
UserWarning: Failed to standardize VSGAGSYQ1T: not a valid amino acid sequence.
  warnings.warn(
/home/yutanagano/Projects/tidytcells/src/tidytcells/aa/_standardize.py:80:
```

```
UserWarning: Failed to standardize VVNGONNDMR: not a valid amino acid sequence.
  warnings.warn(
/home/yutanagano/Projects/tidytcells/src/tidytcells/aa/_standardize.py:80:
UserWarning: Failed to standardize VVNIPNDYXLS: not a valid amino acid sequence.
  warnings.warn(
/home/yutanagano/Projects/tidytcells/src/tidytcells/aa/_standardize.py:80:
UserWarning: Failed to standardize VVSQGYSTIJ: not a valid amino acid sequence.
  warnings.warn(
/home/yutanagano/Projects/tidytcells/src/tidytcells/aa/_standardize.py:80:
UserWarning: Failed to standardize CAMRVSGGSNAKLTFG : not a valid amino acid
sequence.
  warnings.warn(
/home/yutanagano/Projects/tidytcells/src/tidytcells/aa/_standardize.py:80:
UserWarning: Failed to standardize CAXRGGSEKLVF: not a valid amino acid
sequence.
  warnings.warn(
/home/yutanagano/Projects/tidytcells/src/tidytcells/aa/_standardize.py:80:
UserWarning: Failed to standardize CAVERGL##GSQGNLIF: not a valid amino acid
sequence.
  warnings.warn(
/home/yutanagano/Projects/tidytcells/src/tidytcells/aa/_standardize.py:80:
UserWarning: Failed to standardize CAVEDRGEKHXSCL: not a valid amino acid
sequence.
  warnings.warn(
/home/yutanagano/Projects/tidytcells/src/tidytcells/aa/_standardize.py:80:
UserWarning: Failed to standardize CAASSWS*#GLTF: not a valid amino acid
sequence.
  warnings.warn(
/home/yutanagano/Projects/tidytcells/src/tidytcells/aa/_standardize.py:80:
UserWarning: Failed to standardize VQQAQ#YGGSQGNLIF: not a valid amino acid
sequence.
  warnings.warn(
/home/yutanagano/Projects/tidytcells/src/tidytcells/aa/_standardize.py:80:
UserWarning: Failed to standardize CAIELQAR##: not a valid amino acid sequence.
  warnings.warn(
/home/yutanagano/Projects/tidytcells/src/tidytcells/aa/_standardize.py:80:
UserWarning: Failed to standardize CTPG#SQGNLIF: not a valid amino acid
sequence.
  warnings.warn(
/home/yutanagano/Projects/tidytcells/src/tidytcells/aa/_standardize.py:80:
UserWarning: Failed to standardize CI##GGADGLTF: not a valid amino acid
sequence.
  warnings.warn(
/home/yutanagano/Projects/tidytcells/src/tidytcells/aa/_standardize.py:80:
UserWarning: Failed to standardize CAEKARSMVXNYQLIW: not a valid amino acid
sequence.
  warnings.warn(
/home/yutanagano/Projects/tidytcells/src/tidytcells/aa/_standardize.py:80:
```

```
UserWarning: Failed to standardize CAVSGGNYGVXQGNLIF: not a valid amino acid
sequence.
  warnings.warn(
/home/yutanagano/Projects/tidytcells/src/tidytcells/aa/_standardize.py:80:
UserWarning: Failed to standardize CCEGLM#AGTALIF: not a valid amino acid
sequence.
  warnings.warn(
/home/yutanagano/Projects/tidytcells/src/tidytcells/aa/_standardize.py:80:
UserWarning: Failed to standardize CI#GGGADGLTF: not a valid amino acid
sequence.
  warnings.warn(
/home/yutanagano/Projects/tidytcells/src/tidytcells/aa/_standardize.py:80:
UserWarning: Failed to standardize CIVRVLGIVEVATIN#TF: not a valid amino acid
sequence.
  warnings.warn(
/home/yutanagano/Projects/tidytcells/src/tidytcells/aa/_standardize.py:80:
UserWarning: Failed to standardize VQQYSS#QAGTALIF: not a valid amino acid
sequence.
  warnings.warn(
/home/yutanagano/Projects/tidytcells/src/tidytcells/aa/_standardize.py:80:
UserWarning: Failed to standardize XEXXGGXXRXXX##: not a valid amino acid
sequence.
  warnings.warn(
/home/yutanagano/Projects/tidytcells/src/tidytcells/aa/_standardize.py:80:
UserWarning: Failed to standardize CIVSVLG#SGGSNYKLTF: not a valid amino acid
sequence.
  warnings.warn(
/home/yutanagano/Projects/tidytcells/src/tidytcells/aa/_standardize.py:80:
UserWarning: Failed to standardize CTSVQQAL#GGSQGNLIF: not a valid amino acid
sequence.
  warnings.warn(
/home/yutanagano/Projects/tidytcells/src/tidytcells/aa/_standardize.py:80:
UserWarning: Failed to standardize CIVRQLGTGAVVTIN*#F: not a valid amino acid
sequence.
  warnings.warn(
/home/yutanagano/Projects/tidytcells/src/tidytcells/aa/_standardize.py:80:
UserWarning: Failed to standardize CAVED#ISSGSARQLTF: not a valid amino acid
sequence.
  warnings.warn(
/home/yutanagano/Projects/tidytcells/src/tidytcells/aa/_standardize.py:80:
UserWarning: Failed to standardize VL*#GGGADGLTF: not a valid amino acid
sequence.
  warnings.warn(
/home/yutanagano/Projects/tidytcells/src/tidytcells/aa/_standardize.py:80:
UserWarning: Failed to standardize VQQDLGY##GGTSYGKLTF: not a valid amino acid
sequence.
  warnings.warn(
/home/yutanagano/Projects/tidytcells/src/tidytcells/aa/_standardize.py:80:
```

```
UserWarning: Failed to standardize VL*VLPG#ALNF: not a valid amino acid
sequence.
  warnings.warn(
/home/yutanagano/Projects/tidytcells/src/tidytcells/aa/_standardize.py:80:
UserWarning: Failed to standardize CAVND#NTDKLIF: not a valid amino acid
sequence.
  warnings.warn(
/home/yutanagano/Projects/tidytcells/src/tidytcells/aa/_standardize.py:80:
UserWarning: Failed to standardize CAGLGGT#GGSNYKLTF: not a valid amino acid
sequence.
  warnings.warn(
/home/yutanagano/Projects/tidytcells/src/tidytcells/aa/_standardize.py:80:
UserWarning: Failed to standardize CAPQTRRLATTVS*#W: not a valid amino acid
sequence.
  warnings.warn(
/home/yutanagano/Projects/tidytcells/src/tidytcells/aa/_standardize.py:80:
UserWarning: Failed to standardize CAVSGPS#TDSWGKLQF: not a valid amino acid
sequence.
  warnings.warn(
/home/yutanagano/Projects/tidytcells/src/tidytcells/aa/_standardize.py:80:
UserWarning: Failed to standardize CAVSAAX#AGNKLTF: not a valid amino acid
sequence.
  warnings.warn(
/home/yutanagano/Projects/tidytcells/src/tidytcells/aa/_standardize.py:80:
UserWarning: Failed to standardize CAVSA#KAAGNKLTF: not a valid amino acid
sequence.
  warnings.warn(
/home/yutanagano/Projects/tidytcells/src/tidytcells/aa/_standardize.py:80:
UserWarning: Failed to standardize CIVRV#NSNSGYALNF: not a valid amino acid
sequence.
  warnings.warn(
/home/yutanagano/Projects/tidytcells/src/tidytcells/aa/_standardize.py:80:
UserWarning: Failed to standardize CATLCPQ#NKLTF: not a valid amino acid
sequence.
  warnings.warn(
/home/yutanagano/Projects/tidytcells/src/tidytcells/aa/_standardize.py:80:
UserWarning: Failed to standardize VQQAG#SNYKLTF: not a valid amino acid
sequence.
  warnings.warn(
/home/yutanagano/Projects/tidytcells/src/tidytcells/aa/_standardize.py:80:
UserWarning: Failed to standardize CISP*SGSSNTGKLIF: not a valid amino acid
sequence.
  warnings.warn(
/home/yutanagano/Projects/tidytcells/src/tidytcells/aa/_standardize.py:80:
UserWarning: Failed to standardize CIXXL*NDIGENMFLI: not a valid amino acid
sequence.
  warnings.warn(
/home/yutanagano/Projects/tidytcells/src/tidytcells/aa/_standardize.py:80:
```

```
UserWarning: Failed to standardize CIVRVA#SGNTPLVF: not a valid amino acid
sequence.
  warnings.warn(
/home/yutanagano/Projects/tidytcells/src/tidytcells/aa/_standardize.py:80:
UserWarning: Failed to standardize CAG#YGNKLVF: not a valid amino acid sequence.
  warnings.warn(
/home/yutanagano/Projects/tidytcells/src/tidytcells/aa/_standardize.py:80:
UserWarning: Failed to standardize CAXSGGGSYQLTF: not a valid amino acid
sequence.
  warnings.warn(
/home/yutanagano/Projects/tidytcells/src/tidytcells/aa/_standardize.py:80:
UserWarning: Failed to standardize CALPP#YNFNKFYF: not a valid amino acid
sequence.
  warnings.warn(
/home/yutanagano/Projects/tidytcells/src/tidytcells/aa/_standardize.py:80:
UserWarning: Failed to standardize CAVS#SNFGNEKLTF: not a valid amino acid
sequence.
  warnings.warn(
/home/yutanagano/Projects/tidytcells/src/tidytcells/aa/_standardize.py:80:
UserWarning: Failed to standardize VL*#DSSYKLIF: not a valid amino acid
sequence.
  warnings.warn(
/home/yutanagano/Projects/tidytcells/src/tidytcells/aa/_standardize.py:80:
UserWarning: Failed to standardize CADS#ARQLTF: not a valid amino acid sequence.
  warnings.warn(
/home/yutanagano/Projects/tidytcells/src/tidytcells/aa/_standardize.py:80:
UserWarning: Failed to standardize CAASGXGTYKYIF: not a valid amino acid
sequence.
  warnings.warn(
/home/yutanagano/Projects/tidytcells/src/tidytcells/aa/_standardize.py:80:
UserWarning: Failed to standardize VILGV#QKVTF: not a valid amino acid sequence.
  warnings.warn(
/home/yutanagano/Projects/tidytcells/src/tidytcells/aa/_standardize.py:80:
UserWarning: Failed to standardize CVVSAAG#GGGADGLTF: not a valid amino acid
sequence.
  warnings.warn(
/home/yutanagano/Projects/tidytcells/src/tidytcells/aa/_standardize.py:80:
UserWarning: Failed to standardize CVVNGMDSSYKLXF: not a valid amino acid
sequence.
  warnings.warn(
/home/yutanagano/Projects/tidytcells/src/tidytcells/aa/_standardize.py:80:
UserWarning: Failed to standardize CAVDTGNQFYF : not a valid amino acid
sequence.
  warnings.warn(
/home/yutanagano/Projects/tidytcells/src/tidytcells/aa/_standardize.py:80:
UserWarning: Failed to standardize CILXTDSWGKLQF: not a valid amino acid
sequence.
  warnings.warn(
```

```
/home/yutanagano/Projects/tidytcells/src/tidytcells/aa/_standardize.py:80:
UserWarning: Failed to standardize CAVSPXYALNFG: not a valid amino acid
sequence.
  warnings.warn(
/home/yutanagano/Projects/tidytcells/src/tidytcells/aa/_standardize.py:80:
UserWarning: Failed to standardize RARVSATSTDTQ#: not a valid amino acid
sequence.
  warnings.warn(
/home/yutanagano/Projects/tidytcells/src/tidytcells/aa/_standardize.py:80:
UserWarning: Failed to standardize RARDLRELXEQY: not a valid amino acid
sequence.
  warnings.warn(
/home/yutanagano/Projects/tidytcells/src/tidytcells/aa/_standardize.py:80:
UserWarning: Failed to standardize ASXKRD*RGGLQ*A#F: not a valid amino acid
sequence.
  warnings.warn(
/home/yutanagano/Projects/tidytcells/src/tidytcells/aa/_standardize.py:80:
UserWarning: Failed to standardize SAQXTPRPVSS#: not a valid amino acid
sequence.
  warnings.warn(
/home/yutanagano/Projects/tidytcells/src/tidytcells/aa/_standardize.py:80:
UserWarning: Failed to standardize RANGAR#EQY: not a valid amino acid sequence.
  warnings.warn(
/home/yutanagano/Projects/tidytcells/src/tidytcells/aa/_standardize.py:80:
UserWarning: Failed to standardize RXGDRGTDTQ#: not a valid amino acid sequence.
  warnings.warn(
/home/yutanagano/Projects/tidytcells/src/tidytcells/aa/_standardize.py:80:
UserWarning: Failed to standardize ASSLAGRPQIR##: not a valid amino acid
sequence.
  warnings.warn(
/home/yutanagano/Projects/tidytcells/src/tidytcells/aa/_standardize.py:80:
UserWarning: Failed to standardize ASXEGQGLGGWVNXLT: not a valid amino acid
sequence.
  warnings.warn(
/home/yutanagano/Projects/tidytcells/src/tidytcells/aa/_standardize.py:80:
UserWarning: Failed to standardize AXXEGRGWALGPTAX#: not a valid amino acid
sequence.
  warnings.warn(
/home/yutanagano/Projects/tidytcells/src/tidytcells/aa/_standardize.py:80:
UserWarning: Failed to standardize ASSGXGGQPQH: not a valid amino acid sequence.
  warnings.warn(
/home/yutanagano/Projects/tidytcells/src/tidytcells/aa/_standardize.py:80:
UserWarning: Failed to standardize AS*GQGQETQN: not a valid amino acid sequence.
  warnings.warn(
/home/yutanagano/Projects/tidytcells/src/tidytcells/aa/_standardize.py:80:
UserWarning: Failed to standardize ARQGD*RKXDTQ#: not a valid amino acid
sequence.
  warnings.warn(
```

```
/home/yutanagano/Projects/tidytcells/src/tidytcells/aa/_standardize.py:80:
UserWarning: Failed to standardize ASSERGGS#EQY: not a valid amino acid
sequence.
  warnings.warn(
/home/yutanagano/Projects/tidytcells/src/tidytcells/aa/_standardize.py:80:
UserWarning: Failed to standardize RAKLGLEATDTQ#: not a valid amino acid
sequence.
  warnings.warn(
/home/yutanagano/Projects/tidytcells/src/tidytcells/aa/_standardize.py:80:
UserWarning: Failed to standardize VPAIIRGGGXYEEY: not a valid amino acid
sequence.
  warnings.warn(
/home/yutanagano/Projects/tidytcells/src/tidytcells/aa/_standardize.py:80:
UserWarning: Failed to standardize ASSLAGQTPDTQ#: not a valid amino acid
sequence.
  warnings.warn(
/home/yutanagano/Projects/tidytcells/src/tidytcells/aa/_standardize.py:80:
UserWarning: Failed to standardize VPSVPRQE#QPQHF: not a valid amino acid
sequence.
  warnings.warn(
/home/yutanagano/Projects/tidytcells/src/tidytcells/aa/_standardize.py:80:
UserWarning: Failed to standardize ARSWEDTQX: not a valid amino acid sequence.
  warnings.warn(
/home/yutanagano/Projects/tidytcells/src/tidytcells/aa/_standardize.py:80:
UserWarning: Failed to standardize SAPSGPP#NYGYT: not a valid amino acid
sequence.
  warnings.warn(
/home/yutanagano/Projects/tidytcells/src/tidytcells/aa/_standardize.py:80:
UserWarning: Failed to standardize CATDVINDY*LSF: not a valid amino acid
sequence.
  warnings.warn(
/home/yutanagano/Projects/tidytcells/src/tidytcells/aa/_standardize.py:80:
UserWarning: Failed to standardize CAAY#SGTYKYIF: not a valid amino acid
sequence.
  warnings.warn(
/home/yutanagano/Projects/tidytcells/src/tidytcells/aa/_standardize.py:80:
UserWarning: Failed to standardize VL*V#AGGTSYGKLTF: not a valid amino acid
sequence.
  warnings.warn(
/home/yutanagano/Projects/tidytcells/src/tidytcells/aa/_standardize.py:80:
UserWarning: Failed to standardize CATLC#AGNKLTF: not a valid amino acid
sequence.
  warnings.warn(
/home/yutanagano/Projects/tidytcells/src/tidytcells/aa/_standardize.py:80:
UserWarning: Failed to standardize CAVTRW#GGYQKVTF: not a valid amino acid
sequence.
  warnings.warn(
/home/yutanagano/Projects/tidytcells/src/tidytcells/aa/_standardize.py:80:
```

```
UserWarning: Failed to standardize ILPXSWGKL: not a valid amino acid sequence.
  warnings.warn(
/home/yutanagano/Projects/tidytcells/src/tidytcells/aa/_standardize.py:80:
UserWarning: Failed to standardize CVVNNNXDMRF: not a valid amino acid sequence.
  warnings.warn(
/home/yutanagano/Projects/tidytcells/src/tidytcells/aa/_standardize.py:80:
UserWarning: Failed to standardize XVXNREDKLVF: not a valid amino acid sequence.
  warnings.warn(
/home/yutanagano/Projects/tidytcells/src/tidytcells/aa/_standardize.py:80:
UserWarning: Failed to standardize AWKFRDSTYEOY: not a valid amino acid
sequence.
  warnings.warn(
/home/yutanagano/Projects/tidytcells/src/tidytcells/aa/_standardize.py:80:
UserWarning: Failed to standardize ASSITWSGGGAOGIETQY: not a valid amino acid
sequence.
  warnings.warn(
/home/yutanagano/Projects/tidytcells/src/tidytcells/aa/_standardize.py:80:
UserWarning: Failed to standardize ASSLGOQDLVYANTGELF: not a valid amino acid
sequence.
  warnings.warn(
/home/yutanagano/Projects/tidytcells/src/tidytcells/aa/_standardize.py:80:
UserWarning: Failed to standardize ASSLOQGGQETQY: not a valid amino acid
sequence.
  warnings.warn(
/home/yutanagano/Projects/tidytcells/src/tidytcells/aa/_standardize.py:80:
UserWarning: Failed to standardize CASSLRTRTDTQYX: not a valid amino acid
sequence.
  warnings.warn(
/home/yutanagano/Projects/tidytcells/src/tidytcells/aa/_standardize.py:80:
UserWarning: Failed to standardize CASSILGWSEAFX: not a valid amino acid
sequence.
  warnings.warn(
/home/yutanagano/Projects/tidytcells/src/tidytcells/aa/_standardize.py:80:
UserWarning: Failed to standardize CSARTGDRTEAFX: not a valid amino acid
sequence.
  warnings.warn(
/home/yutanagano/Projects/tidytcells/src/tidytcells/aa/_standardize.py:80:
UserWarning: Failed to standardize CALQDXNTGEXFF: not a valid amino acid
sequence.
  warnings.warn(
```

[ ]:
```
num_junction_successes = junctions["corrected"].notna().sum()
num_junction_total = len(junctions)

where_junction_changed = junctions[junctions["junction"] !=␣
 ↪junctions["corrected"]]
```

```
num_successful_junction_changes = where_junction_changed["corrected"].notna().
 ↪sum()
num_junction_changes = len(where_junction_changed)

print(f"total number of IMGT-compliant junctions:                ␣
 ↪{num_tr_successes}")
print(f"original number of unique junctions:                     {num_tr_total}")
print(f"fraction of junctions standardized:                       {num_tr_successes␣
 ↪/ num_tr_total}\n")

print(f"number of junctions originally IMGT-noncompliant:         {num_tr_changes}")
print(f"number of IMGT-noncompliant junctions fixed:             ␣
 ↪{num_successful_tr_changes}")
print(f"fraction of IMGT-noncompliant junctions standardized:␣
 ↪{num_successful_tr_changes / num_tr_changes}")
```

```
total number of IMGT-compliant junctions:            2127
original number of unique junctions:                 2225
fraction of junctions standardized:                  0.9559550561797753

number of junctions originally IMGT-noncompliant:    1217
number of IMGT-noncompliant junctions fixed:         1137
fraction of IMGT-noncompliant junctions standardized: 0.934264585045193
```

[ ]:
```
junctions.to_csv("junctions.csv", index=False)
```

1.5 Test epitope standardization

[ ]:
```
epitopes["corrected"] = epitopes["epitope"].map(tidytcells.aa.standardize)
```

```
/home/yutanagano/Projects/tidytcells/src/tidytcells/aa/_standardize.py:80:
UserWarning: Failed to standardize LLFGFPVYV + SCM(F5): not a valid amino acid
sequence.
  warnings.warn(
/home/yutanagano/Projects/tidytcells/src/tidytcells/aa/_standardize.py:80:
UserWarning: Failed to standardize 1-O-(alpha-D-
galactosyl)-N-hexacosanoylphytosphingosine: not a valid amino acid sequence.
  warnings.warn(
/home/yutanagano/Projects/tidytcells/src/tidytcells/aa/_standardize.py:80:
UserWarning: Failed to standardize
5-(2-oxopropylideneamino)-6-D-ribitylaminouracil: not a valid amino acid
sequence.
  warnings.warn(
/home/yutanagano/Projects/tidytcells/src/tidytcells/aa/_standardize.py:80:
UserWarning: Failed to standardize ASQKRPSQRH + ACET(A1): not a valid amino acid
sequence.
  warnings.warn(
/home/yutanagano/Projects/tidytcells/src/tidytcells/aa/_standardize.py:80:
```

```
UserWarning: Failed to standardize LLFGKPVYV + SCM(K5): not a valid amino acid
sequence.
  warnings.warn(
/home/yutanagano/Projects/tidytcells/src/tidytcells/aa/_standardize.py:80:
UserWarning: Failed to standardize LLFGFAVYV + SCM(F5): not a valid amino acid
sequence.
  warnings.warn(
/home/yutanagano/Projects/tidytcells/src/tidytcells/aa/_standardize.py:80:
UserWarning: Failed to standardize LLFGFPVFV + SCM(F5): not a valid amino acid
sequence.
  warnings.warn(
/home/yutanagano/Projects/tidytcells/src/tidytcells/aa/_standardize.py:80:
UserWarning: Failed to standardize 1-O-(4-deoxy-alpha-D-xylo-
hexopyranosyl)-N-hexacosanoylsphinganine: not a valid amino acid sequence.
  warnings.warn(
/home/yutanagano/Projects/tidytcells/src/tidytcells/aa/_standardize.py:80:
UserWarning: Failed to standardize 1-O-(alpha-D-
galactopyranosyl)-N-icosa-11,14-dienoylphytosphingosine: not a valid amino acid
sequence.
  warnings.warn(
/home/yutanagano/Projects/tidytcells/src/tidytcells/aa/_standardize.py:80:
UserWarning: Failed to standardize 1-O-(3-deoxy-alpha-D-
galactopyranosyl)-N-hexacosanoylsphinganine: not a valid amino acid sequence.
  warnings.warn(
/home/yutanagano/Projects/tidytcells/src/tidytcells/aa/_standardize.py:80:
UserWarning: Failed to standardize 1-O-(alpha-D-
glucopyranosyl)-N-icosa-11,14-dienoylphytosphingosine: not a valid amino acid
sequence.
  warnings.warn(
/home/yutanagano/Projects/tidytcells/src/tidytcells/aa/_standardize.py:80:
UserWarning: Failed to standardize 1-O-(alpha-D-
galactopyranosyl)-N-tetracosanylphytosphingosine: not a valid amino acid
sequence.
  warnings.warn(
/home/yutanagano/Projects/tidytcells/src/tidytcells/aa/_standardize.py:80:
UserWarning: Failed to standardize 1-O-(alpha-D-
glucopyranosyl)-N-tetracosanylphytosphingosine: not a valid amino acid sequence.
  warnings.warn(
/home/yutanagano/Projects/tidytcells/src/tidytcells/aa/_standardize.py:80:
UserWarning: Failed to standardize 1-O-(alpha-D-
galactopyranosyl)-N-tetracosanyl-2-aminononane-1,3,4-triol: not a valid amino
acid sequence.
  warnings.warn(
/home/yutanagano/Projects/tidytcells/src/tidytcells/aa/_standardize.py:80:
UserWarning: Failed to standardize 1-O-(4-deoxy-alpha-D-xylo-
hexopyranosyl)-N-hexacosanoylphytosphingosine: not a valid amino acid sequence.
  warnings.warn(
/home/yutanagano/Projects/tidytcells/src/tidytcells/aa/_standardize.py:80:
```

```
UserWarning: Failed to standardize N-hexacosanoylisoglobotriaosyl ceramide: not
a valid amino acid sequence.
  warnings.warn(
/home/yutanagano/Projects/tidytcells/src/tidytcells/aa/_standardize.py:80:
UserWarning: Failed to standardize alpha-D-galactosyl-(1->4)-beta-D-
galactosyl-(1->4)-beta-D-glucosyl-N-hexacosanoylceramide: not a valid amino acid
sequence.
  warnings.warn(
/home/yutanagano/Projects/tidytcells/src/tidytcells/aa/_standardize.py:80:
UserWarning: Failed to standardize beta-D-galactosyl-(1->4)-beta-D-
glucosyl-N-(docosanoyl)sphingosine: not a valid amino acid sequence.
  warnings.warn(
/home/yutanagano/Projects/tidytcells/src/tidytcells/aa/_standardize.py:80:
UserWarning: Failed to standardize beta-D-
galactosyl-N-(tetracosanoyl)sphingosine: not a valid amino acid sequence.
  warnings.warn(
/home/yutanagano/Projects/tidytcells/src/tidytcells/aa/_standardize.py:80:
UserWarning: Failed to standardize [3)alpha-L-Rhap-(1->2)-alpha-D-
Galp-(1->3)-alpha-D-GlcpNAc-(1->3)-alpha-L-Rhap(1->]4: not a valid amino acid
sequence.
  warnings.warn(
/home/yutanagano/Projects/tidytcells/src/tidytcells/aa/_standardize.py:80:
UserWarning: Failed to standardize 1-elaidoyl-sn-glycero-3-phosphocholine: not a
valid amino acid sequence.
  warnings.warn(
/home/yutanagano/Projects/tidytcells/src/tidytcells/aa/_standardize.py:80:
UserWarning: Failed to standardize 2-O-oleoyl-3-O-palmitoyl-1-O-alpha-D-
galactosyl-sn-glycerol: not a valid amino acid sequence.
  warnings.warn(
/home/yutanagano/Projects/tidytcells/src/tidytcells/aa/_standardize.py:80:
UserWarning: Failed to standardize 1-O-(alpha-D-
galactopyranuronosyl)-N-tetradecanoyldihydrosphingosine: not a valid amino acid
sequence.
  warnings.warn(
/home/yutanagano/Projects/tidytcells/src/tidytcells/aa/_standardize.py:80:
UserWarning: Failed to standardize 1-O-(alpha-D-
galactopyranosyl)-N-hexacosanoyl-D-xylo-phytosphingosine: not a valid amino acid
sequence.
  warnings.warn(
/home/yutanagano/Projects/tidytcells/src/tidytcells/aa/_standardize.py:80:
UserWarning: Failed to standardize (1R)-1,5-anhydro-1-[(3S,4S,5R)-3-
(hexacosanoylamino)-4,5-dihydroxynonadecyl]-D-galactitol: not a valid amino acid
sequence.
  warnings.warn(
/home/yutanagano/Projects/tidytcells/src/tidytcells/aa/_standardize.py:80:
UserWarning: Failed to standardize 1-O-(6-deoxy-6-benzamido-alpha-D-
galactopyranosyl)-N-hexacosanoylphytosphingosine: not a valid amino acid
sequence.
```

```
  warnings.warn(
/home/yutanagano/Projects/tidytcells/src/tidytcells/aa/_standardize.py:80:
UserWarning: Failed to standardize 1-O-{6-deoxy-6-[N'-(1-naphthyl)ureido]-alpha-
D-galactopyranosyl}-N-hexacosanoylphytosphingosine: not a valid amino acid
sequence.
  warnings.warn(
/home/yutanagano/Projects/tidytcells/src/tidytcells/aa/_standardize.py:80:
UserWarning: Failed to standardize alpha-D-
galactosyl-N-(hexacosanoyl)-11,12-methylene-C22-sphinganine: not a valid amino
acid sequence.
  warnings.warn(
/home/yutanagano/Projects/tidytcells/src/tidytcells/aa/_standardize.py:80:
UserWarning: Failed to standardize HS44: not a valid amino acid sequence.
  warnings.warn(
/home/yutanagano/Projects/tidytcells/src/tidytcells/aa/_standardize.py:80:
UserWarning: Failed to standardize 2-palmitoyl-1-cis-vaccenoyl-3-alpha-D-
galactosyl-sn-glycerol: not a valid amino acid sequence.
  warnings.warn(
/home/yutanagano/Projects/tidytcells/src/tidytcells/aa/_standardize.py:80:
UserWarning: Failed to standardize 1-palmitoyl-2-cis-vaccenoyl-3-alpha-D-
glucosyl-sn-glycerol: not a valid amino acid sequence.
  warnings.warn(
/home/yutanagano/Projects/tidytcells/src/tidytcells/aa/_standardize.py:80:
UserWarning: Failed to standardize 1-O-[6-O-(4-pyridylcarbamoyl)-alpha-D-
galactopyranosyl]-N-hexacosanoylphytosphingosine: not a valid amino acid
sequence.
  warnings.warn(
/home/yutanagano/Projects/tidytcells/src/tidytcells/aa/_standardize.py:80:
UserWarning: Failed to standardize 1-O-[6-O-(1-naphthylcarbamoyl)-alpha-D-
galactopyranosyl]-N-hexacosanoylphytosphingosine: not a valid amino acid
sequence.
  warnings.warn(
/home/yutanagano/Projects/tidytcells/src/tidytcells/aa/_standardize.py:80:
UserWarning: Failed to standardize 1-O-{6-O-[(4-chlorophenyl)carbamoyl]-alpha-D-
galactopyranosyl}-N-hexacosanoylphytosphingosine: not a valid amino acid
sequence.
  warnings.warn(
/home/yutanagano/Projects/tidytcells/src/tidytcells/aa/_standardize.py:80:
UserWarning: Failed to standardize 1-O-(alpha-D-
galactopyranosyl)-N-[11-(4-fluorophenyl)undecanoyl]phytosphingosine: not a valid
amino acid sequence.
  warnings.warn(
/home/yutanagano/Projects/tidytcells/src/tidytcells/aa/_standardize.py:80:
UserWarning: Failed to standardize GCK127: not a valid amino acid sequence.
  warnings.warn(
/home/yutanagano/Projects/tidytcells/src/tidytcells/aa/_standardize.py:80:
UserWarning: Failed to standardize GCK152: not a valid amino acid sequence.
  warnings.warn(
```

```
/home/yutanagano/Projects/tidytcells/src/tidytcells/aa/_standardize.py:80:
UserWarning: Failed to standardize alpha-D-
galactosyl-N-[(4Z)-9-(2-decylcyclopropyl)non-4-enoyl]phytosphingosine: not a
valid amino acid sequence.
  warnings.warn(
/home/yutanagano/Projects/tidytcells/src/tidytcells/aa/_standardize.py:80:
UserWarning: Failed to standardize N-[(2S,3S,4R)-1-(alpha-D-
galactosyloxy)-3,4-dihydroxyoctadecan-2-yl]hexacosanethioamide: not a valid
amino acid sequence.
  warnings.warn(
/home/yutanagano/Projects/tidytcells/src/tidytcells/aa/_standardize.py:80:
UserWarning: Failed to standardize 1-(3-O-sulfo-beta-D-
galactosyl)-N-[(15Z)-tetracos-15-enoyl]sphingosine: not a valid amino acid
sequence.
  warnings.warn(
/home/yutanagano/Projects/tidytcells/src/tidytcells/aa/_standardize.py:80:
UserWarning: Failed to standardize 1-(3-O-sulfo-beta-D-
galactosyl)-N-tetracosanoylsphingosine: not a valid amino acid sequence.
  warnings.warn(
/home/yutanagano/Projects/tidytcells/src/tidytcells/aa/_standardize.py:80:
UserWarning: Failed to standardize 1-(3-O-sulfo-beta-D-galactosyl)sphingosine:
not a valid amino acid sequence.
  warnings.warn(
/home/yutanagano/Projects/tidytcells/src/tidytcells/aa/_standardize.py:80:
UserWarning: Failed to standardize 6-deoxy-D-glucos-6-yl corynomycolate: not a
valid amino acid sequence.
  warnings.warn(
/home/yutanagano/Projects/tidytcells/src/tidytcells/aa/_standardize.py:80:
UserWarning: Failed to standardize mycolic acid: not a valid amino acid
sequence.
  warnings.warn(
/home/yutanagano/Projects/tidytcells/src/tidytcells/aa/_standardize.py:80:
UserWarning: Failed to standardize glucose 6-monomycolate (C36): not a valid
amino acid sequence.
  warnings.warn(
/home/yutanagano/Projects/tidytcells/src/tidytcells/aa/_standardize.py:80:
UserWarning: Failed to standardize beta-D-galactosyl-N-(nervonoyl)sphingosine:
not a valid amino acid sequence.
  warnings.warn(
/home/yutanagano/Projects/tidytcells/src/tidytcells/aa/_standardize.py:80:
UserWarning: Failed to standardize reduced
6-(hydroxymethyl)-8-(1-D-ribityl)lumazine: not a valid amino acid sequence.
  warnings.warn(
/home/yutanagano/Projects/tidytcells/src/tidytcells/aa/_standardize.py:80:
UserWarning: Failed to standardize 7-hydroxy-6-methyl-8-(1-D-ribityl)lumazine:
not a valid amino acid sequence.
  warnings.warn(
/home/yutanagano/Projects/tidytcells/src/tidytcells/aa/_standardize.py:80:
```

```
UserWarning: Failed to standardize 6-(hydroxymethyl)-8-(1-D-ribityl)lumazine:
not a valid amino acid sequence.
  warnings.warn(
/home/yutanagano/Projects/tidytcells/src/tidytcells/aa/_standardize.py:80:
UserWarning: Failed to standardize
N(6)-[(2-amino-4-oxo-3,4-dihydropteridin-6-yl)methyl]-D-lysine: not a valid
amino acid sequence.
  warnings.warn(
/home/yutanagano/Projects/tidytcells/src/tidytcells/aa/_standardize.py:80:
UserWarning: Failed to standardize 5-amino-6-(D-ribitylamino)uracil: not a valid
amino acid sequence.
  warnings.warn(
/home/yutanagano/Projects/tidytcells/src/tidytcells/aa/_standardize.py:80:
UserWarning: Failed to standardize
5-(2-oxoethylideneamino)-6-D-ribitylaminouracil: not a valid amino acid
sequence.
  warnings.warn(
/home/yutanagano/Projects/tidytcells/src/tidytcells/aa/_standardize.py:80:
UserWarning: Failed to standardize 6-formylpterin: not a valid amino acid
sequence.
  warnings.warn(
/home/yutanagano/Projects/tidytcells/src/tidytcells/aa/_standardize.py:80:
UserWarning: Failed to standardize N(2)-acetyl-6-formylpterin: not a valid amino
acid sequence.
  warnings.warn(
/home/yutanagano/Projects/tidytcells/src/tidytcells/aa/_standardize.py:80:
UserWarning: Failed to standardize diclofenac: not a valid amino acid sequence.
  warnings.warn(
/home/yutanagano/Projects/tidytcells/src/tidytcells/aa/_standardize.py:80:
UserWarning: Failed to standardize 2-hydroxy-1-naphthaldehyde: not a valid amino
acid sequence.
  warnings.warn(
/home/yutanagano/Projects/tidytcells/src/tidytcells/aa/_standardize.py:80:
UserWarning: Failed to standardize 5-hydroxydiclofenac: not a valid amino acid
sequence.
  warnings.warn(
/home/yutanagano/Projects/tidytcells/src/tidytcells/aa/_standardize.py:80:
UserWarning: Failed to standardize 3-formylsalicylic acid: not a valid amino
acid sequence.
  warnings.warn(
/home/yutanagano/Projects/tidytcells/src/tidytcells/aa/_standardize.py:80:
UserWarning: Failed to standardize 5-methoxysalicylaldehyde: not a valid amino
acid sequence.
  warnings.warn(
/home/yutanagano/Projects/tidytcells/src/tidytcells/aa/_standardize.py:80:
UserWarning: Failed to standardize 2,4-diaminopteridine-6-carbaldehyde: not a
valid amino acid sequence.
  warnings.warn(
```

```
/home/yutanagano/Projects/tidytcells/src/tidytcells/aa/_standardize.py:80:
UserWarning: Failed to standardize LQPFPQPQLPYPQ + DEAM(Q8): not a valid amino
acid sequence.
  warnings.warn(
/home/yutanagano/Projects/tidytcells/src/tidytcells/aa/_standardize.py:80:
UserWarning: Failed to standardize PFPQPQLPYPQPQ + DEAM(Q6): not a valid amino
acid sequence.
  warnings.warn(
/home/yutanagano/Projects/tidytcells/src/tidytcells/aa/_standardize.py:80:
UserWarning: Failed to standardize 1,2-dioleoyl-3-palmitoylglycerol: not a valid
amino acid sequence.
  warnings.warn(
/home/yutanagano/Projects/tidytcells/src/tidytcells/aa/_standardize.py:80:
UserWarning: Failed to standardize palmitoleyl alcohol: not a valid amino acid
sequence.
  warnings.warn(
/home/yutanagano/Projects/tidytcells/src/tidytcells/aa/_standardize.py:80:
UserWarning: Failed to standardize lauryl palmitoleate: not a valid amino acid
sequence.
  warnings.warn(
/home/yutanagano/Projects/tidytcells/src/tidytcells/aa/_standardize.py:80:
UserWarning: Failed to standardize squalene: not a valid amino acid sequence.
  warnings.warn(
/home/yutanagano/Projects/tidytcells/src/tidytcells/aa/_standardize.py:80:
UserWarning: Failed to standardize methyl palmitoleate: not a valid amino acid
sequence.
  warnings.warn(
/home/yutanagano/Projects/tidytcells/src/tidytcells/aa/_standardize.py:80:
UserWarning: Failed to standardize palmitoleic acid: not a valid amino acid
sequence.
  warnings.warn(
/home/yutanagano/Projects/tidytcells/src/tidytcells/aa/_standardize.py:80:
UserWarning: Failed to standardize beryllium atom: not a valid amino acid
sequence.
  warnings.warn(
/home/yutanagano/Projects/tidytcells/src/tidytcells/aa/_standardize.py:80:
UserWarning: Failed to standardize C32 phosphomycoketide: not a valid amino acid
sequence.
  warnings.warn(
/home/yutanagano/Projects/tidytcells/src/tidytcells/aa/_standardize.py:80:
UserWarning: Failed to standardize
N-[(15Z)-tetracosenoyl]sphing-4-enine-1-phosphocholine: not a valid amino acid
sequence.
  warnings.warn(
/home/yutanagano/Projects/tidytcells/src/tidytcells/aa/_standardize.py:80:
UserWarning: Failed to standardize 1-O-oleoyl-sn-glycero-3-phosphocholine: not a
valid amino acid sequence.
  warnings.warn(
```

```
/home/yutanagano/Projects/tidytcells/src/tidytcells/aa/_standardize.py:80:
UserWarning: Failed to standardize oleic acid: not a valid amino acid sequence.
  warnings.warn(
/home/yutanagano/Projects/tidytcells/src/tidytcells/aa/_standardize.py:80:
UserWarning: Failed to standardize 1-stearoyl-2-oleoyl-sn-
glycero-3-phosphocholine: not a valid amino acid sequence.
  warnings.warn(
/home/yutanagano/Projects/tidytcells/src/tidytcells/aa/_standardize.py:80:
UserWarning: Failed to standardize SGQGSFQPSQQNP + DEAM(Q3, Q11): not a valid
amino acid sequence.
  warnings.warn(
/home/yutanagano/Projects/tidytcells/src/tidytcells/aa/_standardize.py:80:
UserWarning: Failed to standardize PQPQLPYPQPQ + DEAM(Q4, Q11): not a valid
amino acid sequence.
  warnings.warn(
/home/yutanagano/Projects/tidytcells/src/tidytcells/aa/_standardize.py:80:
UserWarning: Failed to standardize FPQPQQPFPWQP + DEAM(Q5): not a valid amino
acid sequence.
  warnings.warn(
/home/yutanagano/Projects/tidytcells/src/tidytcells/aa/_standardize.py:80:
UserWarning: Failed to standardize HCLGKWLGHPDKF + ACET(C2): not a valid amino
acid sequence.
  warnings.warn(
/home/yutanagano/Projects/tidytcells/src/tidytcells/aa/_standardize.py:80:
UserWarning: Failed to standardize HSLGKWLGHPDKF + GLYC(H1): not a valid amino
acid sequence.
  warnings.warn(
/home/yutanagano/Projects/tidytcells/src/tidytcells/aa/_standardize.py:80:
UserWarning: Failed to standardize KAVYNFATM + MCM(F6): not a valid amino acid
sequence.
  warnings.warn(
/home/yutanagano/Projects/tidytcells/src/tidytcells/aa/_standardize.py:80:
UserWarning: Failed to standardize KAVYNFATM + MCM(N5): not a valid amino acid
sequence.
  warnings.warn(
/home/yutanagano/Projects/tidytcells/src/tidytcells/aa/_standardize.py:80:
UserWarning: Failed to standardize ASQYRPSQR + ACET(A1): not a valid amino acid
sequence.
  warnings.warn(
/home/yutanagano/Projects/tidytcells/src/tidytcells/aa/_standardize.py:80:
UserWarning: Failed to standardize ASQKRPSQR + ACET(A1): not a valid amino acid
sequence.
  warnings.warn(
/home/yutanagano/Projects/tidytcells/src/tidytcells/aa/_standardize.py:80:
UserWarning: Failed to standardize ASQYRPSQRHG + ACET(A1): not a valid amino
acid sequence.
  warnings.warn(
/home/yutanagano/Projects/tidytcells/src/tidytcells/aa/_standardize.py:80:
```

```
UserWarning: Failed to standardize ASQARPSQRHG + ACET(A1): not a valid amino
acid sequence.
  warnings.warn(
/home/yutanagano/Projects/tidytcells/src/tidytcells/aa/_standardize.py:80:
UserWarning: Failed to standardize ASQKRPSQRHG + ACET(A1): not a valid amino
acid sequence.
  warnings.warn(
/home/yutanagano/Projects/tidytcells/src/tidytcells/aa/_standardize.py:80:
UserWarning: Failed to standardize ASQARPSQR + ACET(A1): not a valid amino acid
sequence.
  warnings.warn(
/home/yutanagano/Projects/tidytcells/src/tidytcells/aa/_standardize.py:80:
UserWarning: Failed to standardize ASQKRPSQR + AMID(A1, R9): not a valid amino
acid sequence.
  warnings.warn(
/home/yutanagano/Projects/tidytcells/src/tidytcells/aa/_standardize.py:80:
UserWarning: Failed to standardize ASQYRPSQR + AMID(A1, R9): not a valid amino
acid sequence.
  warnings.warn(
/home/yutanagano/Projects/tidytcells/src/tidytcells/aa/_standardize.py:80:
UserWarning: Failed to standardize ASQKRPSQRSK + ACET(A1): not a valid amino
acid sequence.
  warnings.warn(
/home/yutanagano/Projects/tidytcells/src/tidytcells/aa/_standardize.py:80:
UserWarning: Failed to standardize AGQFRPSQR + SCM(G2): not a valid amino acid
sequence.
  warnings.warn(
/home/yutanagano/Projects/tidytcells/src/tidytcells/aa/_standardize.py:80:
UserWarning: Failed to standardize AGQARPSQR + SCM(G2): not a valid amino acid
sequence.
  warnings.warn(
/home/yutanagano/Projects/tidytcells/src/tidytcells/aa/_standardize.py:80:
UserWarning: Failed to standardize ATEGRVRVNSAYQDK + CITR(R7): not a valid amino
acid sequence.
  warnings.warn(
/home/yutanagano/Projects/tidytcells/src/tidytcells/aa/_standardize.py:80:
UserWarning: Failed to standardize ATEGRVRVNSAYQDK + CITR(R5, R7): not a valid
amino acid sequence.
  warnings.warn(
/home/yutanagano/Projects/tidytcells/src/tidytcells/aa/_standardize.py:80:
UserWarning: Failed to standardize ATEGRVRVNSAYQDK + CITR(R5): not a valid amino
acid sequence.
  warnings.warn(
/home/yutanagano/Projects/tidytcells/src/tidytcells/aa/_standardize.py:80:
UserWarning: Failed to standardize alpha-D-galactosyl-N-
tetradecanoylsphinganine: not a valid amino acid sequence.
  warnings.warn(
/home/yutanagano/Projects/tidytcells/src/tidytcells/aa/_standardize.py:80:
```

```
UserWarning: Failed to standardize alpha-D-glucuronosylceramide: not a valid
amino acid sequence.
  warnings.warn(
/home/yutanagano/Projects/tidytcells/src/tidytcells/aa/_standardize.py:80:
UserWarning: Failed to standardize 1-O-[alpha-D-galactosyl-(1->2)-alpha-D-
galactosyl]-N-hexacosanoylphytosphingosine: not a valid amino acid sequence.
  warnings.warn(
/home/yutanagano/Projects/tidytcells/src/tidytcells/aa/_standardize.py:80:
UserWarning: Failed to standardize phosphatidylinositol tetramannoside: not a
valid amino acid sequence.
  warnings.warn(
/home/yutanagano/Projects/tidytcells/src/tidytcells/aa/_standardize.py:80:
UserWarning: Failed to standardize 1-hexadecanoyl-2-(9Z-octadecenoyl)-sn-
glycero-3-phospho-D-myo-inositol: not a valid amino acid sequence.
  warnings.warn(
/home/yutanagano/Projects/tidytcells/src/tidytcells/aa/_standardize.py:80:
UserWarning: Failed to standardize ESLKISQAVHAAHAEINEAGRAAAAAK + GLYC(K27): not
a valid amino acid sequence.
  warnings.warn(
/home/yutanagano/Projects/tidytcells/src/tidytcells/aa/_standardize.py:80:
UserWarning: Failed to standardize MLYQHLLPL + OX(M1): not a valid amino acid
sequence.
  warnings.warn(
/home/yutanagano/Projects/tidytcells/src/tidytcells/aa/_standardize.py:80:
UserWarning: Failed to standardize VITAFTEGLK + GLYC(T6): not a valid amino acid
sequence.
  warnings.warn(
/home/yutanagano/Projects/tidytcells/src/tidytcells/aa/_standardize.py:80:
UserWarning: Failed to standardize methoxy mycolic acid: not a valid amino acid
sequence.
  warnings.warn(
/home/yutanagano/Projects/tidytcells/src/tidytcells/aa/_standardize.py:80:
UserWarning: Failed to standardize keto mycolic acid: not a valid amino acid
sequence.
  warnings.warn(
/home/yutanagano/Projects/tidytcells/src/tidytcells/aa/_standardize.py:80:
UserWarning: Failed to standardize
(21E)-3-hydroxy-35-[(icosan-2-yl)oxy]-35-oxo-2-pentacosylpentatriacont-21-enoic
acid: not a valid amino acid sequence.
  warnings.warn(
/home/yutanagano/Projects/tidytcells/src/tidytcells/aa/_standardize.py:80:
UserWarning: Failed to standardize alpha-mycolic acid: not a valid amino acid
sequence.
  warnings.warn(
/home/yutanagano/Projects/tidytcells/src/tidytcells/aa/_standardize.py:80:
UserWarning: Failed to standardize (2R)-2-[(1R)-1-hydroxy-16-{(1R,2S)-2-
[(17R,18R)-17-methoxy-18-methylhexatriacontyl]cyclopropyl}hexadecyl]hexacosanoic
acid: not a valid amino acid sequence.
```

```
  warnings.warn(
/home/yutanagano/Projects/tidytcells/src/tidytcells/aa/_standardize.py:80:
UserWarning: Failed to standardize 1-[(9Z)-octadec-9-enoyl]-2-hexadecanoyl-sn-
glycero-3-phospho-(1'-sn-glycerol): not a valid amino acid sequence.
  warnings.warn(
/home/yutanagano/Projects/tidytcells/src/tidytcells/aa/_standardize.py:80:
UserWarning: Failed to standardize
{1-O-hexadecanoyl-2-O-[(Z)-octadec-9-enoyl]-sn-glycero-3-phospho}serine: not a
valid amino acid sequence.
  warnings.warn(
/home/yutanagano/Projects/tidytcells/src/tidytcells/aa/_standardize.py:80:
UserWarning: Failed to standardize 1-hexadecanoyl-2-(9Z-octadecenoyl)-sn-
glycero-3-phosphocholine: not a valid amino acid sequence.
  warnings.warn(
/home/yutanagano/Projects/tidytcells/src/tidytcells/aa/_standardize.py:80:
UserWarning: Failed to standardize 1-hexadecanoyl-2-(9Z-octadecenoyl)-sn-
glycero-3-phosphoethanolamine zwitterion: not a valid amino acid sequence.
  warnings.warn(
/home/yutanagano/Projects/tidytcells/src/tidytcells/aa/_standardize.py:80:
UserWarning: Failed to standardize 1-palmitoyl-2-oleoyl-sn-glycero-3-phosphate:
not a valid amino acid sequence.
  warnings.warn(
/home/yutanagano/Projects/tidytcells/src/tidytcells/aa/_standardize.py:80:
UserWarning: Failed to standardize 6-deoxy-D-glucos-6-yl (2R,3R)-corynomycolate:
not a valid amino acid sequence.
  warnings.warn(
/home/yutanagano/Projects/tidytcells/src/tidytcells/aa/_standardize.py:80:
UserWarning: Failed to standardize QQYPSGQGSFQPSQQNPQ + DEAM(Q7, Q15): not a
valid amino acid sequence.
  warnings.warn(
/home/yutanagano/Projects/tidytcells/src/tidytcells/aa/_standardize.py:80:
UserWarning: Failed to standardize 1-O-(alpha-D-
galactosyl)-N-[(15Z)-tetracos-15-enoyl]phytosphingosine: not a valid amino acid
sequence.
  warnings.warn(
/home/yutanagano/Projects/tidytcells/src/tidytcells/aa/_standardize.py:80:
UserWarning: Failed to standardize 1-O-(6-acetamido-6-deoxy-alpha-D-
galactosyl)-N-[(15Z)-tetracos-15-enoyl]phytosphingosine: not a valid amino acid
sequence.
  warnings.warn(
/home/yutanagano/Projects/tidytcells/src/tidytcells/aa/_standardize.py:80:
UserWarning: Failed to standardize LEQLESIINFEKLAAAAAK + GLYC(K19): not a valid
amino acid sequence.
  warnings.warn(
/home/yutanagano/Projects/tidytcells/src/tidytcells/aa/_standardize.py:80:
UserWarning: Failed to standardize 2,4-dinitrophenyl group: not a valid amino
acid sequence.
  warnings.warn(
```

```
/home/yutanagano/Projects/tidytcells/src/tidytcells/aa/_standardize.py:80:
UserWarning: Failed to standardize 1-O-(1-O-hexadecanoyl-2-O-heptadecanoyl-sn-
glycero-3-phosphono)-1D-myo-inositol: not a valid amino acid sequence.
  warnings.warn(
/home/yutanagano/Projects/tidytcells/src/tidytcells/aa/_standardize.py:80:
UserWarning: Failed to standardize 1-O-[6-O-(3-phenylpropanoyl)-alpha-D-
galactopyranosyl]-N-hexacosanoylsphinganine: not a valid amino acid sequence.
  warnings.warn(
/home/yutanagano/Projects/tidytcells/src/tidytcells/aa/_standardize.py:80:
UserWarning: Failed to standardize beryllium sulfate: not a valid amino acid
sequence.
  warnings.warn(
/home/yutanagano/Projects/tidytcells/src/tidytcells/aa/_standardize.py:80:
UserWarning: Failed to standardize GVYATRSSAVRLR + CITR(R6): not a valid amino
acid sequence.
  warnings.warn(
/home/yutanagano/Projects/tidytcells/src/tidytcells/aa/_standardize.py:80:
UserWarning: Failed to standardize alpha-galactosylceramide: not a valid amino
acid sequence.
  warnings.warn(
/home/yutanagano/Projects/tidytcells/src/tidytcells/aa/_standardize.py:80:
UserWarning: Failed to standardize PQPQLPYPQ + DEAM(Q4): not a valid amino acid
sequence.
  warnings.warn(
/home/yutanagano/Projects/tidytcells/src/tidytcells/aa/_standardize.py:80:
UserWarning: Failed to standardize PFPQPQLPY + DEAM(Q6): not a valid amino acid
sequence.
  warnings.warn(
/home/yutanagano/Projects/tidytcells/src/tidytcells/aa/_standardize.py:80:
UserWarning: Failed to standardize ASGNHAAGILTM + AMID(M97): not a valid amino
acid sequence.
  warnings.warn(
/home/yutanagano/Projects/tidytcells/src/tidytcells/aa/_standardize.py:80:
UserWarning: Failed to standardize HGAGNHAAGILTL + AMID(L66): not a valid amino
acid sequence.
  warnings.warn(
/home/yutanagano/Projects/tidytcells/src/tidytcells/aa/_standardize.py:80:
UserWarning: Failed to standardize QLQPFPQPQLPY + DEAM(Q9): not a valid amino
acid sequence.
  warnings.warn(
/home/yutanagano/Projects/tidytcells/src/tidytcells/aa/_standardize.py:80:
UserWarning: Failed to standardize LQPFPQPQLPYPQPQ + DEAM(Q8): not a valid amino
acid sequence.
  warnings.warn(
/home/yutanagano/Projects/tidytcells/src/tidytcells/aa/_standardize.py:80:
UserWarning: Failed to standardize PQQPFPQPQQPFP + DEAM(Q9): not a valid amino
acid sequence.
  warnings.warn(
```

```
/home/yutanagano/Projects/tidytcells/src/tidytcells/aa/_standardize.py:80:
UserWarning: Failed to standardize PIPQQPQPYPQ + DEAM(Q4): not a valid amino
acid sequence.
  warnings.warn(
/home/yutanagano/Projects/tidytcells/src/tidytcells/aa/_standardize.py:80:
UserWarning: Failed to standardize phosphatidylglycerol: not a valid amino acid
sequence.
  warnings.warn(
/home/yutanagano/Projects/tidytcells/src/tidytcells/aa/_standardize.py:80:
UserWarning: Failed to standardize 1,2-dioleoyl-3-alpha-D-galactosyl-sn-
glycerol: not a valid amino acid sequence.
  warnings.warn(
/home/yutanagano/Projects/tidytcells/src/tidytcells/aa/_standardize.py:80:
UserWarning: Failed to standardize glucose 6-monomycolate: not a valid amino
acid sequence.
  warnings.warn(
/home/yutanagano/Projects/tidytcells/src/tidytcells/aa/_standardize.py:80:
UserWarning: Failed to standardize 2-O-palmitoyl-2'-O-sulfo-3-O-[(2E,4S,6S,8S)-
2,4,6,8-tetramethyltriacont-2-enoyl]-alpha,alpha-trehalose: not a valid amino
acid sequence.
  warnings.warn(
/home/yutanagano/Projects/tidytcells/src/tidytcells/aa/_standardize.py:80:
UserWarning: Failed to standardize QPPFSQQQQPVL + DEAM(Q6, Q8): not a valid
amino acid sequence.
  warnings.warn(
/home/yutanagano/Projects/tidytcells/src/tidytcells/aa/_standardize.py:80:
UserWarning: Failed to standardize PFSQQQQPVL + DEAM(Q4, Q6): not a valid amino
acid sequence.
  warnings.warn(
/home/yutanagano/Projects/tidytcells/src/tidytcells/aa/_standardize.py:80:
UserWarning: Failed to standardize K68, D69, H74, A75, L76: not a valid amino
acid sequence.
  warnings.warn(
/home/yutanagano/Projects/tidytcells/src/tidytcells/aa/_standardize.py:80:
UserWarning: Failed to standardize H74, A75, L76, K83, K84: not a valid amino
acid sequence.
  warnings.warn(
/home/yutanagano/Projects/tidytcells/src/tidytcells/aa/_standardize.py:80:
UserWarning: Failed to standardize N-hexadecanoylsphingosine-1-phosphocholine:
not a valid amino acid sequence.
  warnings.warn(
/home/yutanagano/Projects/tidytcells/src/tidytcells/aa/_standardize.py:80:
UserWarning: Failed to standardize phosphatidylglycerol 18:0/18:1: not a valid
amino acid sequence.
  warnings.warn(
/home/yutanagano/Projects/tidytcells/src/tidytcells/aa/_standardize.py:80:
UserWarning: Failed to standardize 1-oleoyl-sn-glycerol 3-phosphate: not a valid
amino acid sequence.
```

```
  warnings.warn(
/home/yutanagano/Projects/tidytcells/src/tidytcells/aa/_standardize.py:80:
UserWarning: Failed to standardize maradolipid: not a valid amino acid sequence.
  warnings.warn(
/home/yutanagano/Projects/tidytcells/src/tidytcells/aa/_standardize.py:80:
UserWarning: Failed to standardize 1-oleoyl-2-palmitoyl-sn-
glycero-3-phosphocholine: not a valid amino acid sequence.
  warnings.warn(
/home/yutanagano/Projects/tidytcells/src/tidytcells/aa/_standardize.py:80:
UserWarning: Failed to standardize phosphatidyl-L-serine: not a valid amino acid
sequence.
  warnings.warn(
/home/yutanagano/Projects/tidytcells/src/tidytcells/aa/_standardize.py:80:
UserWarning: Failed to standardize beta-D-GalpNAc-(1->4)-[alpha-
Neup5Gc-(2->3)]-beta-D-Galp-(1->4)-R group: not a valid amino acid sequence.
  warnings.warn(
/home/yutanagano/Projects/tidytcells/src/tidytcells/aa/_standardize.py:80:
UserWarning: Failed to standardize 1-hexadecanoyl-2-(9Z-octadecenoyl)-sn-
glycero-3-phosphoethanolamine: not a valid amino acid sequence.
  warnings.warn(
/home/yutanagano/Projects/tidytcells/src/tidytcells/aa/_standardize.py:80:
UserWarning: Failed to standardize PIM2: not a valid amino acid sequence.
  warnings.warn(
/home/yutanagano/Projects/tidytcells/src/tidytcells/aa/_standardize.py:80:
UserWarning: Failed to standardize phosphatidylinositol: not a valid amino acid
sequence.
  warnings.warn(
/home/yutanagano/Projects/tidytcells/src/tidytcells/aa/_standardize.py:80:
UserWarning: Failed to standardize ganglioside GM1: not a valid amino acid
sequence.
  warnings.warn(
/home/yutanagano/Projects/tidytcells/src/tidytcells/aa/_standardize.py:80:
UserWarning: Failed to standardize PQQPIPQQPQPYPQQ + DEAM(Q2, Q7): not a valid
amino acid sequence.
  warnings.warn(
/home/yutanagano/Projects/tidytcells/src/tidytcells/aa/_standardize.py:80:
UserWarning: Failed to standardize QPFPQPQQPFPW + DEAM(Q7): not a valid amino
acid sequence.
  warnings.warn(
/home/yutanagano/Projects/tidytcells/src/tidytcells/aa/_standardize.py:80:
UserWarning: Failed to standardize ILDQVPFSV + NLeu(L2): not a valid amino acid
sequence.
  warnings.warn(
/home/yutanagano/Projects/tidytcells/src/tidytcells/aa/_standardize.py:80:
UserWarning: Failed to standardize PQPQLPYPQPQ + DEAM(Q4,Q11): not a valid amino
acid sequence.
  warnings.warn(
/home/yutanagano/Projects/tidytcells/src/tidytcells/aa/_standardize.py:80:
```

```
UserWarning: Failed to standardize GGYRARPAKAAAT + CITR(R4, R6): not a valid
amino acid sequence.
  warnings.warn(
/home/yutanagano/Projects/tidytcells/src/tidytcells/aa/_standardize.py:80:
UserWarning: Failed to standardize GGYRARPAKAAAT + CITR(R6): not a valid amino
acid sequence.
  warnings.warn(
/home/yutanagano/Projects/tidytcells/src/tidytcells/aa/_standardize.py:80:
UserWarning: Failed to standardize carbamazepine-10,11-epoxide: not a valid
amino acid sequence.
  warnings.warn(
/home/yutanagano/Projects/tidytcells/src/tidytcells/aa/_standardize.py:80:
UserWarning: Failed to standardize carbamazepine: not a valid amino acid
sequence.
  warnings.warn(
/home/yutanagano/Projects/tidytcells/src/tidytcells/aa/_standardize.py:80:
UserWarning: Failed to standardize oxcarbazepine: not a valid amino acid
sequence.
  warnings.warn(
/home/yutanagano/Projects/tidytcells/src/tidytcells/aa/_standardize.py:80:
UserWarning: Failed to standardize dapsone: not a valid amino acid sequence.
  warnings.warn(
/home/yutanagano/Projects/tidytcells/src/tidytcells/aa/_standardize.py:80:
UserWarning: Failed to standardize nitroso-dapsone: not a valid amino acid
sequence.
  warnings.warn(
/home/yutanagano/Projects/tidytcells/src/tidytcells/aa/_standardize.py:80:
UserWarning: Failed to standardize 3-alpha-D-
glucuronosyl-2-palmitoyl-1-[(10R)-10-methyloctadecanoyl]-sn-glycerol: not a
valid amino acid sequence.
  warnings.warn(
/home/yutanagano/Projects/tidytcells/src/tidytcells/aa/_standardize.py:80:
UserWarning: Failed to standardize 2-palmitoyl-1-cis-vaccenoyl-3-alpha-D-
glucosyl-sn-glycerol: not a valid amino acid sequence.
  warnings.warn(
/home/yutanagano/Projects/tidytcells/src/tidytcells/aa/_standardize.py:80:
UserWarning: Failed to standardize 1-palmitoyl-2-cis-vaccenoyl-3-alpha-D-
galactosyl-sn-glycerol: not a valid amino acid sequence.
  warnings.warn(
/home/yutanagano/Projects/tidytcells/src/tidytcells/aa/_standardize.py:80:
UserWarning: Failed to standardize 1-alpha-D-
glucuronosyl-N-[(2R)-2-hydroxytridecanoyl]sphinganine: not a valid amino acid
sequence.
  warnings.warn(
/home/yutanagano/Projects/tidytcells/src/tidytcells/aa/_standardize.py:80:
UserWarning: Failed to standardize 3-alpha-D-
glucuronosyl-2-[(10R)-10-methyloctadecanoyl]-1-palmitoyl-sn-glycerol: not a
valid amino acid sequence.
```

```
  warnings.warn(
/home/yutanagano/Projects/tidytcells/src/tidytcells/aa/_standardize.py:80:
UserWarning: Failed to standardize SHLVEALYLVCGERGFFYTPK + CITR(R14): not a
valid amino acid sequence.
  warnings.warn(
/home/yutanagano/Projects/tidytcells/src/tidytcells/aa/_standardize.py:80:
UserWarning: Failed to standardize LLSYFGTPT + METH(Y4): not a valid amino acid
sequence.
  warnings.warn(
/home/yutanagano/Projects/tidytcells/src/tidytcells/aa/_standardize.py:80:
UserWarning: Failed to standardize LLSYFGTPT + SCM(Y4): not a valid amino acid
sequence.
  warnings.warn(
/home/yutanagano/Projects/tidytcells/src/tidytcells/aa/_standardize.py:80:
UserWarning: Failed to standardize 6,7-dimethyl-8-(1-D-ribityl)lumazine: not a
valid amino acid sequence.
  warnings.warn(
```

[ ]:
```
num_epitope_successes = epitopes["corrected"].notna().sum()
num_epitope_total = len(epitopes)

where_epitope_changed = epitopes[epitopes["epitope"] != epitopes["corrected"]]
num_successful_epitope_changes = where_epitope_changed["corrected"].notna().
 ↪sum()
num_epitope_changes = len(where_epitope_changed)

print(f"total number of standardized epitopes: {num_tr_successes}")
print(f"original number of unique epitopes:    {num_tr_total}")
print(f"fraction of epiopes standardized:      {num_tr_successes /␣
 ↪num_tr_total}\n")

print("Currently no capacity to fix nonstandard amino acid sequences")
```

```
total number of standardized epitopes: 2127
original number of unique epitopes:    2225
fraction of epiopes standardized:      0.9559550561797753

Currently no capacity to fix nonstandard amino acid sequences
```

[ ]:
```
epitopes.to_csv("epitopes.csv", index=False)
```

[ ]:
```
```
